# Proactive inhibitory control: A general biasing account^☆^

**DOI:** 10.1016/j.cogpsych.2016.01.004

**Published:** 2016-05

**Authors:** Heike Elchlepp, Aureliu Lavric, Christopher D. Chambers, Frederick Verbruggen

**Affiliations:** a Psychology, College for Life and Environmental Sciences, University of Exeter, UK; b School of Psychology and Cardiff University Brain Research Imaging Centre, Cardiff University, UK

**Keywords:** Proactive control, Response inhibition, Dual-task performance, Biased competition, EEG

## Abstract

Flexible behavior requires a control system that can inhibit actions in response to changes in the environment. Recent studies suggest that people proactively adjust response parameters in anticipation of a stop signal. In three experiments, we tested the hypothesis that proactive inhibitory control involves adjusting both attentional and response settings, and we explored the relationship with other forms of proactive and anticipatory control. Subjects responded to the color of a stimulus. On some trials, an extra signal occurred. The response to this signal depended on the task context subjects were in: in the ‘ignore’ context, they ignored it; in the ‘stop’ context, they had to withhold their response; and in the ‘double-response’ context, they had to execute a secondary response. An analysis of event-related brain potentials for no-signal trials in the stop context revealed that proactive inhibitory control works by biasing the settings of lower-level systems that are involved in stimulus detection, action selection, and action execution. Furthermore, subjects made similar adjustments in the double-response and stop-signal contexts, indicating an overlap between various forms of proactive action control. The results of Experiment 1 also suggest an overlap between proactive inhibitory control and preparatory control in task-switching studies: both require reconfiguration of task-set parameters to bias or alter subordinate processes. We conclude that much of the top-down control in response inhibition tasks takes place before the inhibition signal is presented.

## Highlights

• Most work on proactive inhibitory control (PIC) is descriptive.
• The theoretical accounts focus primarily on response- or motor-related processes.
• We show that PIC biases stimulus detection and response selection.
• We also demonstrate an overlap between various forms of proactive control.
• Based on our findings, we propose a general biasing account for PIC.

## 1 Introduction

At the core of flexible and goal-directed behavior is the ability to replace or withhold planned actions in response to changes in the environment or internal states. Response inhibition receives much attention across research domains, including cognitive, social, developmental, and clinical psychology, cognitive neuroscience, psychopharmacology, and psychiatry (see e.g. Aron, 2011, Bari and Robbins, 2013, Verbruggen et al., 2013, Verbruggen and Logan, 2008). For example, deficiencies in response inhibition have been associated with psychological disorders such as attention deficit/hyperactivity disorder, obsessive–compulsive disorder, substance abuse, pathological gambling, and overeating (Bechara et al., 2006, Chambers et al., 2009, Crews and Boettiger, 2009, de Wit, 2009, Garavan and Stout, 2005, Nigg, 2001, Noël et al., 2013). The study of response inhibition has therefore been a major component in the endeavor to better understand and combat impulsive and compulsive behavior. However, early studies failed to explain *how* responses are stopped when a stop signal is presented (‘reactive inhibitory control’) and they did not acknowledge that successful response inhibition largely depends on advance task preparation (‘proactive inhibitory control’). In recent years, several studies have proposed detailed accounts of the cognitive processes underlying reactive inhibitory control. Here we present a detailed cognitive account of proactive inhibitory control in the stop-signal paradigm.

The stop-signal paradigm is currently one of the most popular paradigms to study response inhibition in the laboratory because it allows researchers to estimate the covert latency of response inhibition: the stop-signal reaction time (SSRT). SSRT has become an established and important marker for reactive inhibitory control on stop-signal trials (Verbruggen et al., 2013). Many researchers use SSRT as a pure measure of reactive inhibitory control, and they attribute (implicitly or explicitly) differences between groups, individuals, and conditions to variation in the effectiveness of suppressing motor output. However, reactive inhibitory control on stop-signal trials involves a chain of processes that results in a response being withheld (e.g., Boucher et al., 2007, Logan et al., 2014, Logan et al., 2015, Salinas and Stanford, 2013, van de Laar et al., 2010, Verbruggen and Logan, 2015, Verbruggen et al., 2014, Verbruggen et al., 2014). More specifically, stopping requires perceptual, decisional (action selection), and motor-related processes to be successful. Consequently, SSRT reflects more than the duration of a single neural motor-related inhibitory process (Verbruggen & Logan, 2015).

Researchers have also used the stop-signal paradigm to study how people adjust their behavior when they are informed that they may have to stop a response in the near future (proactive inhibitory control). Unfortunately, most of the work on proactive inhibitory control is descriptive and relies on general constructs. For example, researchers often equate proactive inhibitory control with ‘response slowing’. Other studies have focused primarily on anticipatory regulation of response activation or motor excitability (for reviews, see Aron, 2011, Stuphorn and Emeric, 2012). Most likely, this response- or motor-related focus stems from the focus on response-related processes in the reactive inhibitory control literature. For example, Verbruggen and Logan (2009a) fitted sequential sampling models to stop-signal data and found that subjects increased the response thresholds when they anticipated a stop signal, resulting in longer go reaction times and higher go accuracy (Verbruggen & Logan, 2009a; see also Logan et al., 2014). Other studies suggest that motor output is proactively modulated when stop signals can occur (e.g., Cai et al., 2011, Lavallee et al., 2014, Lo et al., 2009, Wiecki and Frank, 2013, Wong-Lin et al., 2010).

The present study has three specific aims. First, we aim to provide clear evidence for the idea that proactive inhibitory control involves adjusting both attentional and response parameters. Second, we will test the idea that similar control adjustments are made in situations in which no response inhibition is required. Third, we will test the idea that proactive inhibitory control is similar to the dynamic reconfiguration of task-set parameters in situations in which people have to switch tasks on a trial-by-trial basis. By testing these three ideas in a single study using the same paradigm, we go well beyond previous research on proactive inhibitory control, which has tended to focus on which response settings are adjusted in anticipation of a stop signal. Ultimately, we aim to provide a comprehensive, integrative theoretical account of proactive inhibitory control that is strongly supported by empirical data and grounded in the wider literatures.

First, we propose that proactive inhibitory control involves adjusting attentional settings. This aspect of proactive control has been largely neglected or minimized in the response inhibition literature. For example, Stuphorn and Emeric (2012, p. 5) argued in their review that proactive inhibitory control is mostly related to a regulation of the level of excitability of the motor system. However, recent research highlights the importance of perceptual processes for reactive stopping (as discussed above), and most of SSRT may be occupied by afferent processes (e.g. Boucher et al., 2007, Logan et al., 2014, Salinas and Stanford, 2013). Consequently, it seems plausible that proactive inhibitory control could also involve adjusting attentional settings to enhance detection of the stop signal on signal trials. This idea received some support from studies by Greenhouse and Wessel (2013) and Schevernels et al. (2015). They found that the N1, which is an event-related potential associated with stimulus detection (see below for a detailed discussion), was larger on stop-signal trials when successful stop performance was rewarded. This could indicate that reward-related (proactive) control adjustments could influence signal detection. However, Greenhouse and Wessel (2013) found no N1 differences between successful and unsuccessful signal trials, which led them to conclude that visual attention was not related to stopping successes. Furthermore, these studies focused on stop-signal ERPs, so it is unclear whether the modulated N1 is due to a general motivational effect that influences reactive control performance (Boehler, Schevernels, Hopf, Stoppel, & Krebs, 2014) or to proactive adjustments of attentional parameters. We tried to examine the latter in a stop-signal experiment in which stop signals could occur in the center of the screen or in the periphery (Verbruggen, Stevens, et al., 2014). On half of the trials, perceptual distractors were presented throughout the trial. These distractors had the largest effect on go responses in the non-central signal blocks compared with central-signal blocks and blocks in which no signals could occur. This suggests that subjects widened the attentional focus for detecting the signal in the periphery in the face of interference from distractors. However, this experiment was unconventional in that stop signals (especially in the non-central condition) were harder to detect than in a typical stop-signal experiment, so it is not clear whether biasing attentional selection is an integral component of proactive inhibitory control. Furthermore, we could not test whether subjects also adjusted decisional and motor settings. Therefore, the first aim of the present study is to provide strong support for the idea that proactive inhibitory control involves modulation of all processing stages in the go task, including attentional and response selection. As such, this study goes beyond our previous theoretical work in which the focus was more general, and in which we also failed to acknowledge the role of proactive attentional adjustments of attentional settings in response-inhibition paradigms (Verbruggen, McLaren, et al., 2014).

Second, ‘proactive inhibitory control’ typically refers to strategic adjustments in anticipation of a stop signal, but we propose that subjects make similar proactive control adjustments in anticipation of other acts of control. Indeed, work in other control domains indicates that people can adjust attentional and response parameters when they anticipate certain events to occur (for reviews, see e.g. Braver, 2012, Verbruggen et al., 2014). In interference and working-memory tasks, proactive control involves activation of the relevant task goals, which biases activation in subordinate attentional and working-memory systems (e.g. Braver, 2012, Braver et al., 2007). For example, in a Stroop interference task, activation of the ‘color naming’ goal before a trial would enhance detection of the relevant color feature and reduce interference caused by irrelevant word features on incongruent trials (Braver, 2012). Work in the visual attention domain (for a review, see Carrasco, 2011) has also shown that detection of a stimulus can be enhanced by advance information of its location (Posner, 1980) or other, non-spatial, features, such as shape, color, or direction of motion (Corbetta & Shulman, 2002). These attentional phenomena have been linked to anticipatory activity in the visual cortex and other sensory areas (e.g. Chelazzi et al., 1998, Kastner and Ungerleider, 2000, Luck et al., 1997, Sylvester et al., 2007). Similarly, when the control system predicts a certain action, it can proactively activate the motor network, biasing action selection and reducing the response latency of the anticipated action (e.g. Bestmann, 2012). Thus, there seems to be a conceptual overlap between ‘proactive inhibitory control’ (which involves according to us adjustments of attentional and response settings) and proactive control adjustments in other domains. However, the substantial differences between control tasks make it difficult to directly compare proactive control adjustments in different domains. Therefore, we will test the overlap idea directly by contrasting performance in a stop-signal condition with a performance in a control condition that does not require inhibition of responses, but which is otherwise very similar to the stop condition.

Third, we propose that proactive inhibitory control involves rapidly adjusting task parameters. Most proactive inhibitory control studies have used block-based manipulations to study proactive inhibitory control (contrasting blocks in which no signals could occur with blocks in which signals could occur), but some studies have demonstrated that adjustments can be made on a trial-by-trial basis (e.g. Verbruggen & Logan, 2009a). Similar dynamic adjustments of task parameters have been demonstrated in the *task-switching* paradigm (e.g., Meiran, 1996, Rogers and Monsell, 1995), which requires subjects to switch among two or more tasks (which typically use the same set of stimuli). A robust finding in the task-switching literature is that switching between tasks is associated with a cost, which is typically substantially reduced by providing an opportunity to prepare for the switch (for reviews, see Kiesel et al., 2010, Monsell, 2003, Vandierendonck et al., 2010). When changing task involves shifts of attention among locations or stimulus dimensions, a substantial part of the switch cost has been attributed to (re)setting of attentional selection parameters (Elchlepp et al., 2015, Longman et al., 2013, Longman et al., 2014, Mayr et al., 2013). Similarly, when changing task involves updating the stimulus–response mappings, a substantial part of the switch cost has been attributed to (re)setting the response parameters (Kieffaber et al., 2013, Meiran et al., 2000, Rushworth et al., 2002, Yeung and Monsell, 2003). In Logan and Gordon’s Executive Control of Theory of Visual Attention (ECTVA) model (Logan & Gordon, 2001), the executive system adjusts the parameters of both the visual attention and response selection processes on dual-task or task-switch trials; the adjustment of perceptual selection and response parameters is also at the core of the CARIS task-set modelling framework (Meiran, 2000, Meiran et al., 2008). By doing so, the executive control system can support flexible behavior in multi-task situations. Thus, we propose that proactive inhibitory control can occur on a trial-by-trial basis, and that it is similar to reconfiguring task-set parameters when switching between tasks.

## 2 The present study

Researchers have argued that successful inhibition depends on proactive adjustments of response thresholds and anticipatory suppression of motor activation. However, most of this work offers a (often narrow) response-focused view on ‘proactive inhibitory control’. Here we propose that optimal performance in stop-signal tasks and other response-inhibition paradigms requires finding a balance between focusing on the relevant go stimuli and monitoring the environment for potentially relevant stop signals (i.e. an optimal configuration of attentional settings), and finding a balance between going fast on go trials and stopping when a signal occurs (i.e. an optimal configuration of response settings) (Study Aim 1). Furthermore, we argue that proactive inhibitory control adjustments are similar to adjustments made in other control tasks or situations (Study Aims 2 and 3).

We tested these ideas in three experiments. In each experiment, we use a modified version of the context-cueing task (Verbruggen, Aron, Stevens, & Chambers, 2010), in which subjects performed a color judgment in three possible task contexts (ignore, stop, and double-response respectively). On each trial, the identity of a black string of letters or symbols (e.g. ‘IGNR’ for ignore, ‘XXXX’ for stop, or ‘++++’ for double-response) specified the context; we refer to it as the ‘cue’. When the string turned yellow or green (thus becoming a ‘go stimulus’), subjects initiated a go response (this was their primary task). On a minority of trials, the colored go stimulus turned bold (the ‘signal’) after a variable delay: in the ignore context (signal-ignore trials), subjects had to ignore the signal and execute the go response (i.e. they could always respond); in the stop context (stop-signal trials), subjects were instructed to withhold the primary (go) response; and in the double-response context (double-response-signal trials), subjects had to execute an additional response following the go response by pressing an alternate key. In a previous study (Verbruggen et al., 2010), we used a variant of this task to examine reactive inhibitory control after transcranial magnetic stimulation of the right inferior frontal junction and the right inferior frontal gyrus. We included the double-response condition to test the specificity of the reactive control mechanisms: on both the double-response and stop-signal trials, subjects must detect an extra signal and subsequently select an appropriate (non-dominant) response. A detailed analysis revealed that reactive inhibitory control mechanisms on stop-signal trials overlapped strongly with the cognitive mechanisms underlying the execution of the secondary response on double-response signal trials.^1^

In the present study, we used the context-cueing task to examine proactive inhibitory control. We combined the context-cueing paradigm with event-related potentials (ERPs). ERPs are an online measure of neural activity, with an excellent temporal resolution, providing the opportunity to monitor how proactive inhibitory control modulates attentional and response settings. In order to get a ‘pure’ measure of proactive control (i.e., adjustments in anticipation of an event), we will focus on no-signal trials.

A comparison of no-signal ERPs in the ‘ignore’ and ‘stop’ contexts can reveal to what extent people adjust both attentional and response settings when they expect a stop signal in the near future (Study Aim 1). Our account postulates that people alter the settings of the lower-level perceptual and response systems to find a balance between going fast on no-signal trials and stopping on a signal trial. Therefore, we expect that proactive inhibitory control will influence the processing of the go stimuli, the selection of the appropriate go response, and its execution. In particular, any differences between the ignore and stop contexts regarding the detection of visual stimuli should be reflected in early sensory ERP components such as the N1 and/or Selection Negativity (e.g., Anllo-Vento et al., 1998, Luck and Kappenman, 2012, Potts and Tucker, 2001, Schupp et al., 2007, Vogel and Luck, 2000, Wills et al., 2014, for review). Any differences between the ignore and stop contexts in allocation of attentional resources (e.g., Johnson, 1986, Johnson, 1984, Kramer et al., 1985), in response-selection settings, or both, should be reflected in the P3 (e.g., Verleger, Jaskowski, & Wascher, 2005). Finally, any differences between the contexts in response-execution settings should be reflected in the response-locked LRPs (e.g., Smulders & Miller, 2012).

We included the double-response context to examine the specificity of the proactive control adjustments (Study Aim 2). Based on the overlap in reactive control mechanisms (see above), we predict an overlap between preparing for a stop signal and preparing for a double-response signal. Thus, any changes to no-signal ERP components in the stop context should also be observed in the double-response context.

Third, we also measured an ERP correlate of the setting (adjustment) of task parameters to examine whether proactive control in response-inhibition tasks is similar to preparatory control of a task set when switching between completely different tasks (also referred to as anticipatory ‘task-set reconfiguration’, Lavric et al., 2008, Monsell, 2003) (Study Aim 3). In the most widely used version of the task-switching paradigm, ‘task-cueing’ (Meiran, 1996), a cue presented before stimulus onset specifies the task. When the cue–stimulus interval is sufficiently long, subjects can interpret the cue and reconfigure the task-set parameters in advance of the imperative stimulus (see above). Similarly, in our task subjects could interpret the (black) context cue and adjust processing parameters accordingly prior to the presentation of the (colored) go stimulus. Thus, in addition to using the above-mentioned ERP components that should reflect the application and maintenance of (adjusted) control parameters (N1, SN, P3 and LRP), we were also interested in an ERP correlate of the setting (adjustment) as it unfolds. More specifically, we examined whether the presentation of the context cue was associated with a component typically found in preparation intervals in task-switching studies, namely the posterior switch-related positivity (see Karayanidis et al., 2010, for a review). The magnitude of this switch positivity correlates with successful switching performance, both within and across subjects (Elchlepp et al., 2012, Karayanidis et al., 2011, Lavric et al., 2008). This has led to the conclusion that the posterior switch positivity is an ERP correlate of dynamic and anticipatory task-set reconfiguration (Karayanidis et al., 2010, Karayanidis et al., 2011, Lavric et al., 2008; but see Kang, DiRaddo, Logan, & Woodman, 2014, for a different interpretation of the posterior positivity). If our assumptions about proactive inhibitory control are correct, then switching to a context that potentially requires an act of control should necessitate an adjustment of task parameters, just like when switching to another task. Therefore, we predict that switching to another action context should elicit a posterior positivity (relative to repeating the same context) in the late part of the preparation interval – the interval between the cue and the go stimulus.

## 3 Experiment 1

In Experiment 1, subjects responded to the color of a go stimulus in the primary task (the ‘go’ task). On a minority of the trials, the stimulus turned bold, instructing the subjects to withhold their go response (the ‘stop context’) or to execute a second response (‘the double-response’) context. The context changed on 2/3 of the trials. To examine the consequences of proactive inhibitory control, we focused on four ERP components in the primary go task: the N1, the Selection Negativity (SN), the P3, and the response-locked Lateralised Readiness Potential (LRP). The first two components have been associated with stimulus detection and perceptual (feature) selection, respectively, whereas the latter two have been associated with response selection and response execution, respectively. Finally, to examine the online setting (adjustment) of the parameters and the overlap between proactive inhibitory control and task switching, we focused on the posterior positivity.

### 3.1 Method

#### 3.1.1 Subjects

Thirty-two right-handed adults (25 female) with an average age of 20 (ranging from 18 to 30) were paid £15 for participation in this study. Eleven subjects were replaced, one due to high error rates and ten due to low trial numbers in the ERP averages (please see under ‘EEG and ERP’ section for the numerous exclusion criteria for the no-signal trial analyses; combined with the exclusions due to eye blink and muscle artifacts, these criteria led to an unusually high number of excluded subjects). The subject- and trial-specific exclusion criteria or filters were decided prior to hypothesis testing.

#### 3.1.2 Apparatus, stimuli, paradigm, and procedure

**Fig. 1:**
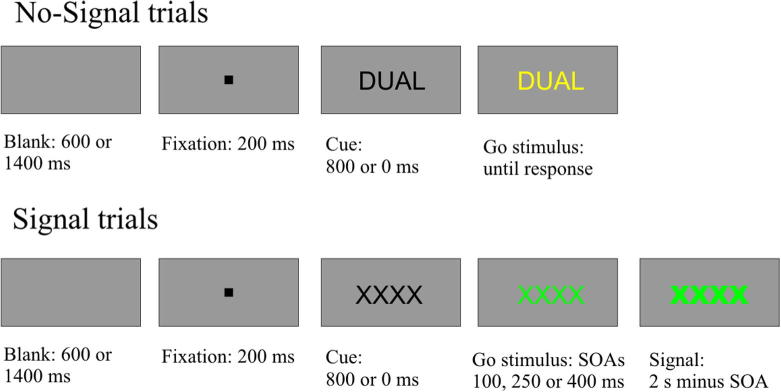
Trial structure and example stimuli.

The task was programmed in E-prime (Schneider, Eschman, & Zuccolotto, 2002). The trial structure and example stimuli are presented in Fig. 1. The stimuli were presented on a TFT monitor against a gray background in Lucinda Console font, size 24, subtending 1.9° visual angle.

There were two preparation conditions, separated into different experimental blocks (see below). In the ‘preparation’ condition (3/4 of the blocks), each trial started with the presentation of a black fixation dot in the center of the screen for 200 ms, followed by a ‘cue’ (a black string of letters or symbols) that specified the relevant context: ‘IGNR’ or ‘====’ indicated the ignore context; ‘HOLD’ or ‘XXXX’ indicated the stop context; ‘DUAL’ or ‘++++’ indicated the double-response context. After 800 ms, the black string turned yellow or green (i.e. the cue became a ‘go stimulus’). The primary task was to respond as quickly and accurately as possible to the color of the go stimulus by pressing ‘c’ or ‘m’ on a QWERTY keyboard with the left or right index finger, respectively. The color-response mapping (e.g. green = m, yellow = c) was counterbalanced across subjects. The color stimulus stayed on the screen until a response was executed. In the ‘no preparation’ condition (1/4 of the blocks) the fixation dot was immediately followed by the colored string (the go stimulus; e.g., ‘HOLD’ in green). This condition was included to determine whether subjects took the opportunity to prepare for the upcoming context in the ‘preparation’ condition: in both conditions the identity of the string specified the context, but only in the ‘preparation’ condition this information was available (in the form of the black cue) in advance of the to-be-identified color.

On 25% of the trials in both preparation conditions, the go stimulus turned bold (signal trials) after one of three intervals (stimulus onset asynchronies or SOAs: 100, 250 or 400 ms). The SOAs were the same for all three contexts, and occurred randomly and with equal probability. In the ignore context, subjects were instructed to ignore the signal and respond to the color of the stimulus only; in the stop context, subjects were instructed to withhold the color response when the signal appeared; in the double-response context, subjects were instructed to respond to the signal by pressing the space bar with the left or right thumb after they responded to the color. Furthermore, subjects were told not to wait for the stop signal to occur and were informed that it would be easy to stop on some trials and difficult or impossible to stop on other trials. For double-response signal trials, they were told to execute the two responses independently, as quickly as possible, and not to group responses. (Subjects using a grouping strategy would select the go response – i.e. ‘c’ or ‘m’ – but then hold it waiting until the second response – i.e. the space-bar – was also ready to be initiated; cf. Ulrich & Miller, 2008). On signal trials, the colored string remained on the screen for 2 s (regardless RT or SOA).

The context changed unpredictably on 2/3 of the trials. The identity of the go stimulus (and that of the cue in the ‘preparation’ condition) was never the same as on the previous trial, even when the context remained the same as on the previous trial (e.g., when the stop context was repeated ‘HOLD’ was always followed by a ‘XXXX’ and vice versa). This was done because in task switching it has been shown that changing the task cue results in a performance cost, even when the task is repeated (e.g., Logan & Bundesen, 2003, and Monsell & Mizon, 2006).

The session started with nine blocks of practice of 49–50 trials each. In the first three blocks subjects practiced each context separately. In the following six blocks they practiced switching between contexts (five preparation blocks and one no-preparation block). The experimental part of the session (during which behavioral and EEG data were collected) comprised 24 blocks, of which 18 contained 49 trials and 6 contained 50 trials: Given the 25% probability of a signal trial, one quarter of the blocks started with a signal trial followed by a filler trial, which were both discarded from the behavioral and EEG analyses (as were all other trials following a signal; see below). Pseudorandom trial sequences were generated subject to the following constraints: the probability of a context switch (2/3) was the same for signal and no-signal trials for each stimulus type (i.e. words or symbol strings); all context switches (ignore-stop, ignore-double, stop-ignore, stop-double, double-ignore, double-stop) were equiprobable; and the probability of a signal was the same for each stimulus type by context combination. A new sequence was generated for each participant whilst enforcing these constraints separately for each preparation condition. No-preparation blocks were interspersed among preparation blocks so that one no-preparation block was followed by three preparation blocks (e.g., nP-P-P-P-nP-P-P-P …). To avoid confounding practice effects with preparation effects, we counterbalanced the starting point of this sequence across subjects (e.g., Subject 1: P-nP-P-P-P …; Subject 2: P-P-nP-P-P-P …).

#### 3.1.3 EEG data acquisition

The electroencephalogram (EEG) was acquired using 64 Ag/AgCl active electrodes (ActiCap, Brain Products, Munich, Germany) connected to BrainAmp amplifiers (Brain Products, Munich, Germany). The EEG was sampled continuously at 500 Hz with a bandpass of 0.016–100 Hz, the reference at Cz and the ground at AFz. There were 62 electrodes on the scalp in an extended 10–20 configuration and one on each earlobe. Their impedances were kept below 10 kΩ.

#### 3.1.4 Analyses

All raw and processed behavioral and EEG data are deposited in the Open Research Exeter data repository (http://hdl.handle.net/10871/19336). We will focus on performance on no-signal trials to get ‘pure’ measures of proactive control (see above); for completeness, we present the signal data in Appendix A.

##### 3.1.4.1 Behavioral analyses

Trials following an incorrect response, trials following a signal trial, and the first trial of each block (which cannot be classified as a context switch or repeat) were excluded from the analyses. Incorrect trials were also excluded from the no-signal RT analysis. No-signal performance was analyzed using univariate analyses of variance with context, context repetition, and preparation condition as within-subject factors. In planned follow-up analyses, we contrasted the stop and ignore contexts to examine proactive inhibitory control adjustments, and the double-response context with the ignore and stop contexts to examine the generality of these adjustments.

##### 3.1.4.2 ERPs

Because the total number of no-preparation trials and the number of signal trials were low, we only analyzed ERPs for no-signal trials in the preparation condition. The EEG was filtered offline with a 20 Hz low-pass (48 dB/oct) and a 0.1 Hz high pass (24 dB/oct) filter, and re-referenced to the linked ears. To correct eye blink artifacts, we ran an Independent Component Analysis (Infomax ICA, Bell & Sejnovski, 1995), implemented in Vision Analyzer (BrainProducts, Munich, Germany). Sixty-three ICA components were obtained from every subject’s EEG (the same as the number of electrodes submitted to ICA). For each subject we excluded on average eight components with characteristic eye-blink and eye-movement topographies and time-courses. Following this ICA-based artifact subtraction, the EEG was segmented into 1800 ms epochs, time-locked to the presentation of the cue and baseline-corrected relative to the average amplitude of the 100 ms preceding the cue. The long segments were further sub-segmented into: −100 ms to 800 ms segments time locked to the presentation of the cue, and −100 ms to 1000 ms time locked to the presentation of the go stimulus. For both ERP segments, we used the same pre-cue baseline (i.e., the 100 ms interval before the cue onset).^2^ For response-locked LRPs, segments were cut from −700 ms preceding the response to 200 ms following the response, and the first 100 ms of that segment (from −700 to −600 ms) were used as a baseline.

Consistent with the behavioral analyses, we excluded from all ERP analyses segments associated with incorrect no-signal trials, segments following errors, segments corresponding to the first trial of a block, signal trials, and trials following a signal. The resulting segments were visually inspected for residual ocular, muscle, movement and other artifacts, and segments containing such artifacts were removed. The remaining EEG segments were averaged for every subject and experimental condition. Subjects’ cue-locked averages contained around 80 trials for context switches and around 40 trials for context repeats (this difference originated from having three contexts with two-thirds context switches and one third context repeats).^3^ Go stimulus-locked ERP averages contained about 60 trials for each context (here we averaged over switch-repeat because there were no effects or interactions involving switching; see Results section).

**Fig. 2:**
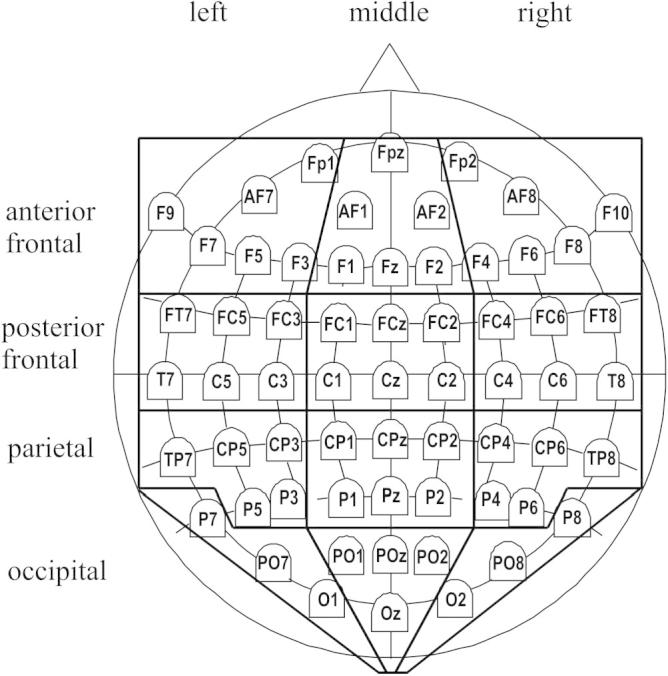
Electrode grouping into regions.

In our previous task-cueing studies, the switch-related positivity emerged around 400 ms after cue onset with maximal amplitudes at 600–800 ms (e.g., Elchlepp et al., 2012, Lavric et al., 2008). For our analysis we therefore averaged amplitudes in the time window between 400 and 800 ms. Given the posterior maximum of the switch positivity, we averaged ERP amplitudes within this interval for groups of electrodes in left, middle and right parietal and occipital regions of the scalp (see Fig. 2) and submitted them to a 3 (laterality) by 2 (region) ANOVA. When components with a relatively broad scalp distribution are investigated, averaging over scalp regions grossly based on cortical anatomy is a common approach in ERP research in general, and ERP task-switching studies in particular (e.g., Elchlepp et al., 2012, Goffaux et al., 2006, Lavric et al., 2008, Poljac and Yeung, 2014, Poulsen et al., 2005). This approach avoids (or reduces the severity) of the problem of electrode selection for the analysis, it improves signal-to-noise ratio via spatial smoothing, and, most importantly, enables tests of differences in scalp distribution (e.g. by using topographic factors in the ANOVA).

As mentioned in Footnote 2, residual cue-related activity could spill over into the ERP segment time-locked to the go stimulus. To avoid such contamination of differences in early perceptual components, we used peak-to-peak measures for the analyses of amplitude differences in the N1 and the Selection Negativity: the N1 amplitude was quantified as the N1-P1 difference, and the amplitude of the Selection Negativity (SN) as the difference between the P2 and the first negative peak following the P2 for each participant and context. There was no reason to expect a lateralization of these early visual components (the stimuli were presented centrally), hence their amplitudes were quantified as peak amplitude in electrodes PO8 and PO7 and subsequently averaged over these electrodes.

Existing analyses of the time-course of the preparation switch-related positivity (by means of temporal PCA, Elchlepp et al., 2012) suggest that the spillover of this effect from the preparation interval should dissipate by the time the P3 rises. Hence, we quantified the amplitude of the P3 as the mean amplitude over the 300–500 ms interval to capture the broad range of this component (instead of the peak-to-peak procedure employed for the N1 and the SN). To capture the typical P3 topography, we averaged amplitudes of electrodes in the posterior frontal, parietal and occipital middle regions of the scalp (see Fig. 2). In the main P3 analysis, amplitudes of all electrodes in these regions were averaged – so as to employ the same statistical test as for the N1 and the SN, enabling correction for the inflation in Type 1 error over all the tests conducted for the three components (N1, SN and P3). However, components from the P3 family can have different scalp distributions (the frontal P3b and the posterior P3b). Hence, we also tested for differences between regions (P3a, P3b) with an ANOVA including the factors context and region (see the Results section).

Finally, we calculated the Lateralised Readiness Potential (LRP), using the standard procedure (e.g., Coles, 1989) implemented in Brain Vision Analyser. Preparing a motor response results in a negative wave over the motor cortex contralateral to the response hand (the Readiness Potential, e.g., Deecke, Grozinger, & Kornhuber, 1976). To isolate this activation and distinguish it from non-lateralised ERP deflections occurring at about the same time (e.g., the P3) we subtracted the amplitude in an electrode positioned over the motor cortex ipsilateral to the responding hand (C4 for right hand and C3 for left hand responses) from the amplitude in a contralateral electrode (C3 for right and C4 for left hand responses) and averaged the results of the left and right hand subtractions. Waveforms were smoothed with a low-pass digital filter (with a high-frequency cutoff at 8 Hz; e.g., De Jong, Coles, & Logan, 1995). Given this LRP derivation procedure, lateralization can only arise once the decision of which hand to use is made. The LRP time-locked to the onset of the response is informative about the duration of response activation and peripheral motor processes and thus provides additional insight to what is learned from the analyses of the components in the go stimulus-locked ERPs (N1, SN, and P3).

We analyzed onset latency, peak amplitude and peak latency of the r-LRP using the “jackknifing” method (Miller, Patterson, & Ulrich, 1998), which has been developed to deal with the difficulties of identifying ERP morphological features and determining their latencies in individual subjects. Instead of using the individual subjects’ ERPs to calculate the standard error, this procedure calculates it based on sub-averages of all subjects but one (e.g., for 32 subjects 32 sub-averages are created, first leaving out subject 1, then subject 2, etc., until subject 32 is left out). Even with the help of jackknifing, accurate estimation of ERP components can be challenging and different onset estimation methods have been found superior in different circumstances (for reviews on detecting the LRP onset, see Miller et al., 1998, and Mordkoff & Gianaros, 2000). We therefore chose two of these methods in an attempt to obtain converging evidence. First, we determined the onset as the time when the r-LRP waveform reached 30% of its peak amplitude. Second, we fitted a bilinear function and used the inflection point between the best-fitting two linear segments as an estimate of the onset (this procedure is described in detail in Appendix B). We applied a strict conjunction criterion: a difference between experimental conditions (contexts) was only conclusive if it was statistically significant in *both* onset analyses.

For statistical analysis, we used *t*-tests to run planned pairwise comparisons of different levels of one factor (i.e., the pairwise contrasts between the experimental contexts) and univariate analyses of variance whenever there was more than one factor and the interaction was tested (i.e., the interaction between the factors context and switch in the analysis of the preparation interval, and the interaction between context and region in the P3 analysis). For the paired samples *t*-tests, we used Cohen’ *d_av_* to calculate the effects sizes (Lakens, 2013) and the Holm–Bonferroni method (Holm, 1979) to control for the inflation of familywise error in multiple comparisons within each of the three ‘families’ of tests: (1) the ERP analysis time-locked to the cue (where multiple comparisons were needed to follow up the ANOVA), (2) the ERP analyses time-locked to the go stimulus (where multiple comparisons were conducted to examine the stimulus-locked components N1, SN and P3), and (3) the ERP analysis time-locked to the response, where multiple tests were required to assess differences in r-LRP latency and amplitude; the correction was applied separately to the subsets of tests conducted on the r-LRP onset latency, peak latency and peak amplitude. For the ANOVAs, significance levels were adjusted using the Huynh–Feldt correction for violations of sphericity (but unadjusted degrees of freedom are reported).

### 3.2 Results and discussion

#### 3.2.1 Behavioral results

Descriptive statistics for no-signal trials (mean RTs and error rates) are shown in Table 1, Table 2, respectively; inferential statistics are given in Table 3.

The signal data are presented in Appendix A.

Consistent with previous work, we found that no-signal RTs were influenced by context (*p* < .001; Table 3). Planned comparisons revealed that mean no-signal RTs were significantly longer in the stop context (789 ms) than in the ignore context (644 ms), *t*(31) = 7.2, *p* < .001, *d_av_* = 1.23. This indicates that subjects adopted a more cautious response strategy in the context in which stop signals could occur. RTs were also longer in the double-response context (726 ms) than in the ignore context (644 ms), *t*(31) = 5.59, *p* < .001, *d_av_* = .67. Thus, the dual-task requirement in the double-response condition also slowed RTs. Finally, RTs were longer in the stop context than in the double-response context, *t*(31) = 6.15, *p* < .001, *d_av_* = .50, indicating that the behavioral effect of proactive control adjustements was most pronounced when subjects anticipated a stop signal.

The effect of advance preparation differed for the three contexts (interaction: *p* < .01, see Table 3): in the ignore and double-response context, RTs were shorter (by about 90 ms) in the preparation condition than in the no-preparation condition, which suggests that subjects encoded the cue and prepared for the upcoming context in the preparation condition. In the stop context, this reduction was much smaller (about 50 ms). In this context, advance preparation could produce slowing as subjects have time to adopt a more cautious response strategy before the stimulus appears (see e.g. Verbruggen & Logan, 2009a). This can also explain why RTs were numerically shorter on context-switch trials than on context-repetition trials in this stop context (Table 1): on repetition trials, subjects have already implemented a more cautious response strategy, resulting in longer reaction times.

#### 3.2.2 ERP results

In this section, we report the analyses of the ERPs for no-signal trials in the preparation condition. First, we report the analyses of the ERPs following the onset of the go stimulus (i.e., the interval after the stimulus changed color) to examine which processing stages in the go task are influenced by proactive inhibitory control (Study Aim 1), and how context-specific these adjustments are (Study Aim 2). Then we examine the preparation interval (i.e. the interval between the presentation of the cue and the go stimulus) where we predicted a posterior positivity when context changed relatively to a context repetition, by analogy with the task-switching literature (Study Aim 3).

##### 3.2.2.1 ERPs following the onset of the go stimulus

To explore possible effects of switching and context, we divided the ERPs into 100 ms bins, from 0 to 700 ms following go stimulus onset. ERPs differed between contexts in all time windows but no reliable interactions with switching were found (all *p*’s > .4). We also tested for potential effects of switch on each ERP component reported below but failed to discover any (these analyses are presented in Appendix C). The lack of evidence for switch effects in the post-stimulus intervals suggests that subjects used the preparation interval effectively to prepare for a switch to the upcoming context, as discussed in more detail below. In the absence of significant interactions with switching, we averaged switch and repeat ERP amplitudes for each context to augment the signal-to-noise ratio for these ERP data.

###### 3.2.2.1.1 N1

**Fig. 3:**
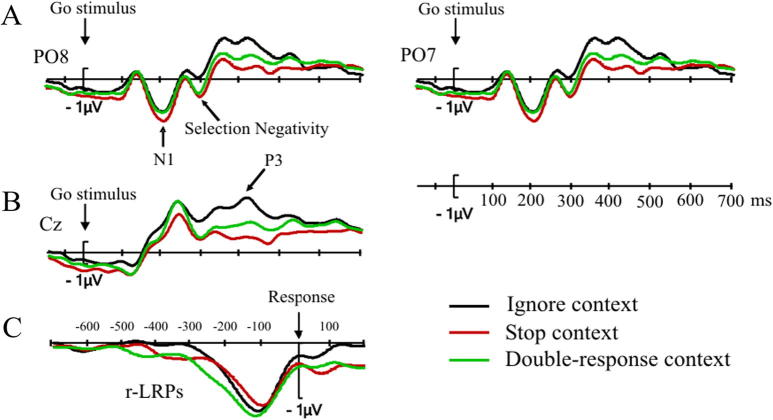
Experiment 1, post go stimulus waveforms for all contexts: A in electrodes PO8 (left) and PO7 (right), B in Cz, C response-locked LRPs.

We use the N1 is a marker of stimulus detection, as it reflects the perception of a visual or auditory stimulus (with different scalp distributions for the two modalities: a posterior scalp distribution for the visual modality, and a central scalp distribution for the auditory modality). For both modalities, previous studies indicate that N1 amplitudes are larger when more attention is directed to perceiving the stimulus (e.g., Luck et al., 1990, Vogel and Luck, 2000, for the visual N1; e.g., Hillyard, Hink, Schwent, & Picton, 1973, for the auditory N1). Grand average N1 amplitudes in electrodes PO8 and PO7 are shown in Fig.3A. The mean N1 amplitude (using the peak-to-peak measure – the N1-P1 difference) was largest in the stop context, −6.6 μV, followed by the double-response context, −6.3 μV, and smallest in the ignore context, −5.9 μV. The difference between the stop and ignore context was significant, *t*(31) = 3.48, *p* < .05, *d_av_* = .24. This suggests that more attention is directed to the color (go) stimulus in the stop context. More specifically, proactive control exerted in the preparation interval may have led to an increased gain in visual cortex in anticipation of the visual stop signal. The differences between the double-response and the ignore context and the stop and double-response contexts were not significant, *t*(31) = 1.94, *p* = .2, *d_av_* = .14, and *t*(31) = 1.68, *p* < .3, *d_av_* = .1, respectively.

###### 3.2.2.1.2 Selection Negativity

The next component to be modulated by context was the Selection Negativity (see Fig.3A for grand average waveforms in electrodes PO7 and PO8), which has been argued to reflect the continued processing of a visual stimulus with the aim of detecting a particular feature (Anllo-Vento and Hillyard, 1996, Potts and Tucker, 2001, Schupp et al., 2007, Wills et al., 2014). The SN amplitude (quantified from the preceding peak, P2, see Method) was significantly larger in the stop context (−3.55 μV) than in the ignore context (−2.57 μV), *t*(31) = 3.23, *p* < .05, *d_av_* = .5, which indicates that subjects monitored for the go stimulus to turn bold. The differences between the double-response (−3.39 μV) and the ignore and stop contexts were not significant, *t*(31) = 2.58, *p* = .07, *d_av_* = .41, and *t*(31) = −0.7, *p* = .5, *d_av_* = .07, respectively.

###### 3.2.2.1.3 P3

The P3 presumably reflects resource allocation during the decision-making process (Johnson, 1984, Johnson and Donchin, 1982, Kramer et al., 1985) and/or linking the decision with the correct response (Verleger et al., 2005). *T*-tests on P3 amplitude showed it to be larger in the ignore context (4.5 μV) than in the stop (3.1 μV) and double-response (3.4 μV) contexts, *t*(31) = −3.92, *p* < .01, *d_av_* = 0.61, and *t*(31) = −4.31, *p* < .01, *d_av_* = 0.46, respectively (see also Fig.3B). The difference between the double-response and stop contexts was not significant, *t*(31) = −1.45, *p* = .3, *d_av_* = 0.16. Appendix D shows the P3 analysis as a function of region. No significant interactions with region were found.

Although the P3 is one of the most widely studied components there is no universal agreement on its functional interpretation. The amplitude of the P3 may be sensitive to stimulus uncertainty (Johnson, 1984, Johnson, 1986) and attentional demands (e.g. Kramer et al., 1985). In our double-response and stop-signal contexts, there was uncertainty if and when a signal would occur, and monitoring for the signal might have drawn attention away from processing the primary task stimulus. Both could have reduced the P3 amplitude (Johnson, 1984, Johnson, 1986, Kramer et al., 1985). Furthermore, Verleger et al. (2005) found that the P3 amplitude was smaller and had a much broader peak (due to more temporal jitter) when the response decision was difficult compared to when it was easy. In Experiment 1, response difficulty could also have contributed to smaller P3 amplitudes in the dual-task contexts in which subjects occasionally had to select an alternative action plan. Thus, our results are in line with these influential accounts of the P3, linking it to stimulus evaluation, attentional resource allocation and early stages of response selection.

###### 3.2.2.1.4 Lateralised Readiness Potential

The r-LRP (Fig.3C) reached 30% of peak amplitude at −271 ms for double-response context, at −238 ms for the stop context, and at −220 ms for the ignore context. The r-LRP onset estimate obtained by bilinear fitting was the earliest (in relation to the response) for the double-response context (−504 ms), followed by the stop context (−432 ms), followed by the ignore context (−316 ms). Jackknifing-based *t*-tests revealed that the two methods of comparing r-LRP onsets converged in finding a significantly earlier onset in the double-response context than in the ignore context (30%, *t*(31) = −3.06, *p* < .05; bilinear, *t*(31) = −5.18, *p* < .001). The remaining comparisons (stop vs. ignore, stop vs. double-response) were both significant in the bilinear analysis, but not in the 30% analysis (for the statistics, see Appendix E).

We also examined the amplitude and latency of the r-LRP peaks. The peak amplitudes for the different contexts were −1.3 μV in the stop context, −1.5 μV in the double-response context, and −1.4 μV in the ignore context. These amplitude differences were not significant (all *p*’s > .1). The peak latencies were −100 ms in the stop context, −120 ms in the double-response context, and −112 ms in the ignore context. These differences were also not significant (all *p*’s > .1).

The r-LRP marks the last stage of action programming; it arises before the movement is executed. The interval between its onset and the response reflects how long it takes to program and implement an action. Previous research indicates that motor output is suppressed when subjects expect a stop-signal to occur (e.g., Cai et al., 2011). The numerical r-LRP onset differences were consistent with these findings: r-LRP onset was earlier (i.e. the interval between the LRP onset and the execution of the response was longer) in the stop- and double-response contexts in than in the ignore context. However, only the double-response vs. ignore difference was significant in both r-LRP analyses. Therefore, these r-LRP onset differences should be interpreted with caution.

##### 3.2.2.2 Preparation interval

**Fig. 4:**
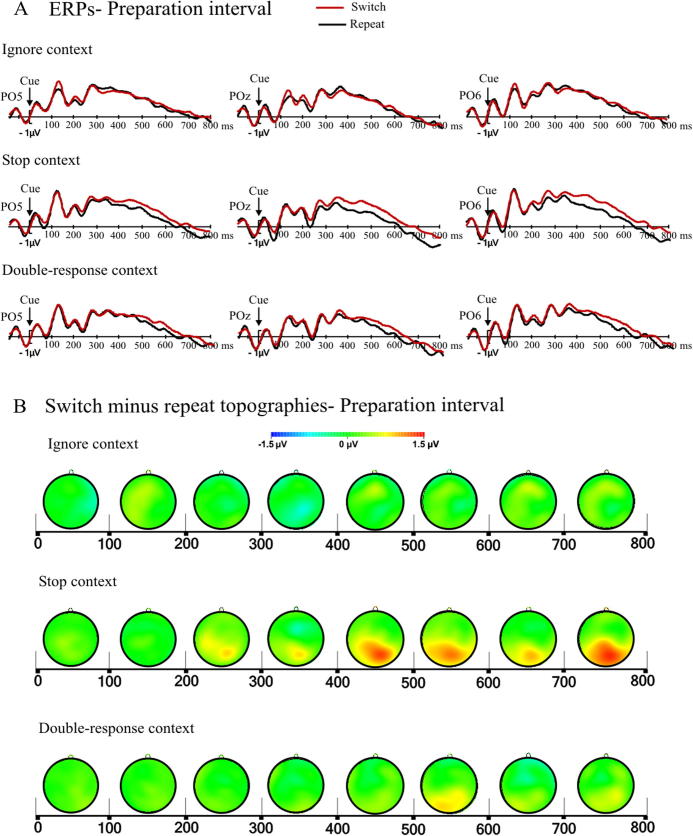
Experiment 1, A waveforms for switch and repeat for all contexts in electrodes PO5, POz and PO6, and B topographies of the switch – repeat differences.

This analysis focused on the effect of preparation for a context after just having performed one or more trials in a different context, or in other words, the effect of switching to a new context compared to repeating the same. ERP studies of task switching consistently find more positive amplitudes on the posterior scalp for switch compared to repeat trials in the later part of the preparation interval (e.g., Karayanidis et al., 2003, Lavric et al., 2008, Nicholson et al., 2005). Fig. 4 shows the waveforms for switch and repeat for all three contexts in representative electrodes, and topographies of the switch-repeat differences. Here we describe only effects or interactions with the factor switch in detail. We found a main effect of switch, *F*(1, 31) = 11.51, *p* < .01, partial *η*^2^ = .271, and a switch by region interaction, *F*(1, 31) = 9.21, *p* < .01, partial *η*^2^ = .229. Although the interaction between switch and context failed to reach significance (*F*(2, 62) = 2.65, *p* = .08, partial *η*^2^ = .079), it was important to determine which context(s) showed statistically reliable effects of switch. We therefore conducted separate analyses to examine the switch-repeat difference in each context: this difference was significant in the stop context, *F*(1, 31) = 11.69, *p* < .01, partial *η*^2^ = .274, but not in the double-response context, *F*(1, 31) = 3.71, *p* = .1, partial *η*^2^ = .107, or the ignore context, *F*(1, 31) = 0.2, *p* = .66, partial *η*^2^ = .006. There were no significant interactions with region in any of the contexts (stop, *p* > .16; double-response, *p* > .6; ignore, *p* > .3).

These findings suggest that when subjects switched to a context in which stop signals could occur, they used the preparation interval to actively prepare for this potential task demand. It seems unlikely that this reflects a response-inhibition specific process because the time-course and scalp distribution of the observed switch positivity closely resembles that of the posterior switch positivity found in ERP task-switching studies^4^ that did not require stopping an already planned response. Instead, it is more consistent with the idea that proactive inhibitory control is similar to preparing to switch tasks. We will come back to this in the General Discussion. This finding also contributes to the debate on whether proactive strategy adjustments can be made on a trial-by-trial basis. For example, in studies by Brown and Steyvers, 2005, Los, 1999, and Strayer and Kramer (1994), subjects did not seem to make use of pre-cues to proactively prepare their response strategies. Other studies found proactive adjustments only under certain conditions, for example when there was a requirement to respond as fast as possible (Gopher et al., 2000, Kleinsorge, 2001, Los, 1999). Our behavioral and electrophysiological results unambiguously show that proactive inhibitory control strategies can be employed on a trial-by-trial basis (see also Verbruggen & Logan, 2009a).

### 3.3 Interim conclusion

Experiment 1 provides clear answers to three main questions raised in the Introduction. First, the analysis of post-go-stimulus ERPs showed that proactive inhibitory control influenced both the perceptual processing of the go stimulus and the selection of the go response on no-signal trials. More specifically, we found differences in the N1 with larger amplitudes in the stop context than in the ignore context, which could reflect increased attention on stimulus processing in the stop context (e.g., Luck, Fan, & Hillyard, 1993). The amplitude of the Selection Negativity, which could be seen as a marker of signal monitoring, was also larger in the stop context than in the ignore context. This suggests a more thorough perceptual analysis of the go stimulus in anticipation of it turning bold. Finally, modulations of the P3 suggest that response-selection processes were modulated by task context. Combined, these results indicate that proactive inhibitory control modulated different processing stages in the go task, which is consistent with the idea that subjects try to balance task demands by adjusting parameters of subordinate processes to enhance detection of the stop signal and to prevent premature go responses (Study Aim 1).

Second, the P3 and r-LRP analyses indicate that subjects also made proactive control adjustments in the double-response task. The analyses of the N1 and the SN also revealed numerical differences between the double-response context and the ignore context, but these failed to reach significance. These results suggest similarities between proactive control in the stop and double-response contexts, but also some (probably quantitative) differences (Study Aim 2).

Third, the marker of task-set reconfiguration found in task-switching ERP studies, the posterior switch positivity, was also present when preparing for a switch to the stop context. This indicates that there is indeed a functional overlap between proactive inhibitory control and task switching, which is consistent with the idea that both involve anticipatory updating of task-set parameters (Study Aim 3).

**Fig. A1:**
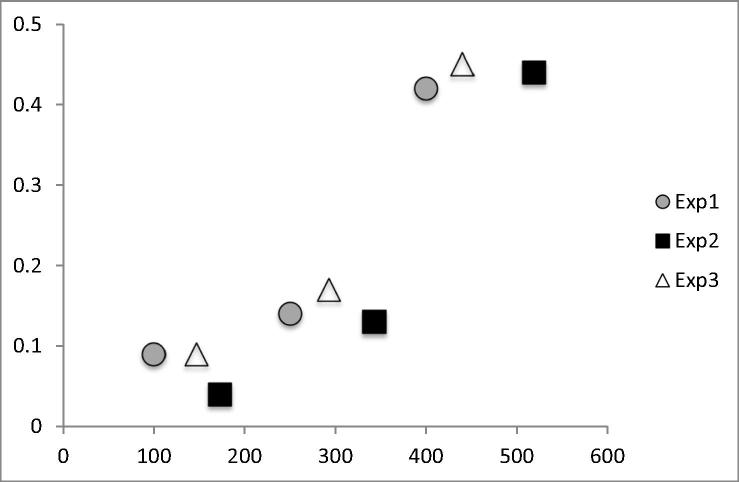
Inhibition functions depict the relation between *p*(respond|signal) (*Y*-axis) and stop-signal delay (*X*-axis).

We should note that the probability of responding on stop-signal trials was lower than in previous stop-signal studies (see Appendix A). This could indicate that subjects simply waited for the signal to occur and that they decided after the longest SOA had elapsed whether to initiate a go response or not. However, a careful analysis of the stop-signal data suggests that subjects engaged in the inhibition of an initiated go response on stop-signal trials. In Fig. A1 in Appendix A, we show inhibition functions for Experiments 1–3. These functions plot the relation between *p*(respond|signal) and the stop signal delay (SSD), and are important theoretically because they reflect the outcome of the race between the go process and the stop process (Verbruggen & Logan, 2009b). The independent horse-race model of Logan and Cowan (1984) assumes that the SSD will influence the relative finishing time of the stop process: when SSD increases, the stop process will start later and therefore, finish later compared to the go process; consequently, response inhibition will succeed less often. The inhibition functions for Experiments 1–3 are consistent with the predictions of the independent race model, and suggest that a stop process was required to withhold the response when a signal occurred. The presence of a stop-P3 in the stop-signal ERPs (see Appendix A) in Experiments 2–3 further indicates that subjects engaged a stopping mechanism on stop-signal trials.

## 4 Experiment 2

In Experiment 2, we further explored the idea that proactive control adjustments modulate both stimulus-detection and response-related processes in both the stop and double-response contexts (Study Aims 1 and 2). The ERP positivity induced by a switch of context, which was our on-line measure of proactive reconfiguration of control parameters in Experiment 1, cannot be unambiguously linked to a specific processing stage. Indeed, in the task-switching domain, the switch positivity has been elicited by changes in both perceptual and response-related task-set components (see Karayanidis et al., 2010, for a discussion). In Experiment 2, we therefore focused on post-stimulus ERP components reflecting the application of control settings in different contexts because these ERP components have the potential to distinguish between processing stages (and the associated parameters). We simplified the design by blocking contexts so that all trials (apart from the first trial of a block, which was excluded from the analyses) were context repetitions.

### 4.1 Method

#### 4.1.1 Subjects

Thirty right-handed adults (19 female) with an average age of 24 (ranging from 18 to 48) were paid £12 for their participation in this study.

#### 4.1.2 Apparatus, stimuli, and procedures

The set-up and paradigm were very similar to those in Experiment 1, except for the differences outlined below. We removed the trial-by-trial switching manipulation, hence one cue/stimulus per context was sufficient, and single characters replaced the strings. The characters ‘=’ (ignore context), ‘+’ (double-response context), and ‘X’ (the stop context) subtended 1.4° visual angle.

Each block of 60 trials consisted of three runs of 20 trials of the same context (e.g., 20 ignore, 20 double-response, 20 stop context trials). The order of the contexts was the same for all 18 blocks in the experiment for a given subject, but was counterbalanced across subjects. In each run of 20 trials, 14 trials were no-signal trials and six were signal trials. Each trial started with a blank screen with a gray background for 500 ms, which was followed by the cue. After 250 ms the cue turned yellow or green (i.e. became the go stimulus). It stayed on the screen until a response was given or 1500 ms had passed. On signal trials the stimulus turned bold after a variable delay (see details for the SOA calculation below). Feedback was provided on every trial. On no-signal, ignore signal, and double-response signal trials, the word “correct” was displayed after correct responses, “wrong key” was displayed after an incorrect key press, and “try to respond faster” was shown if no response was detected within 1500 ms on no-signal and ignore-signal trials or if subjects did not respond in time to the signal on double-response trials (i.e. 1500 ms minus SOA). On stop-signal trials “correct” was displayed after a successful stop, and “try to stop” was shown after a failed stop. The feedback stayed on the screen for 750 ms, after which the next trial started.

In this version of the paradigm, subjects only switched contexts twice in a block of 60 trials (i.e., after a run of 20 trials in one context). Before the first trial of the next context, the screen went blank for 1500 ms. Subjects were told in advance that this indicated the change to the next context (the order of contexts was known to them and they had experienced context changes in the practice session). Nevertheless we excluded the first trial of each run to minimize the effects of context switching and start-up effects (e.g., Allport and Wylie, 2000, Gopher et al., 2000).

Subjects started with a practice session consisting of two blocks, which were identical to the experimental blocks with the exception that SOAs were fixed at 100, 250 and 400 ms. After completion of those blocks, each subject’s mean reaction time of correct no-signal trials was calculated for each context. For each subject, the three SOAs for the rest of the experiment corresponded to 25%, 50% and 75% of their mean RT in the first two practice blocks for a given context. Subjects then performed one more practice block with the individualized SOAs before starting the experimental session.

#### 4.1.3 EEG/ERP analyses

The EEG set-up and pre-processing steps were the same as in Experiment 1, except that there were no analyses of the preparation interval. For no-signal trials, the EEG was cut into segments time locked to the stimulus, from 350 ms preceding to 1000 ms following go stimulus onset. Segments were baseline corrected to the 350–250 ms average preceding the color stimulus onset. This interval was used (instead of the common −100 to 0 ms pre-go-stimulus interval) to avoid the contamination of the baseline by potential differences between contexts elicited by the cue (appearing 250 ms before the go stimulus). As before, we focused on no-signal trials only to test our account of proactive inhibitory control. The ERP averages for each context and subject contained about 150 trials.

### 4.2 Results and discussion

#### 4.2.1 Behavioral results

Consistent with Experiment 1, we focus on no-signal trials only (but see Appendix A for the signal data). Response times for no-signal trials were significantly longer in the stop context (*M* = 807 ms, *sd* = 122 ms) than in the ignore context (*M* = 469 ms, *sd* = 108 ms), *t*(29) = 13.5, *p* < .001, *d_av_* = 2.94, and in the double-response context (*M* = 582 ms, *sd* = 144 ms), *t*(29) = 10.7, *p* < .001, *d_av_* = 1.69. The RT difference between double-response and ignore was also significant, *t*(29) = 5.2, *p* < .001, *d_av_* = 0.9.

The probability of a correct response was higher in the stop context (*M* = 98.8%, *sd* = 1.4%) than in the ignore context (*M* = 96.8%, *sd* = 2.8%), *t*(29) = −4.01, *p* < .001, *d_av_* = 0.971, and in the double-response context (*M* = 98.1%, *sd* = 1.6%), *t*(29) = −2.37, *p* < .05, *d_av_* = 0.431. The difference between the ignore context and the double-response context was also significant, *t*(29) = −4.12, *p* < .001, *d_av_* = 0.638. The number of missed responses was overall very low (too low to warrant inferential statistics): stop context (*M* = 0.8%, *sd* = 1.3%), ignore context (*M* = 0.2%, *sd* = 0.5%), and double-response context (*M* = 0.1%, *sd* = 0.3%).

#### 4.2.2 ERP results

**Fig. 5:**
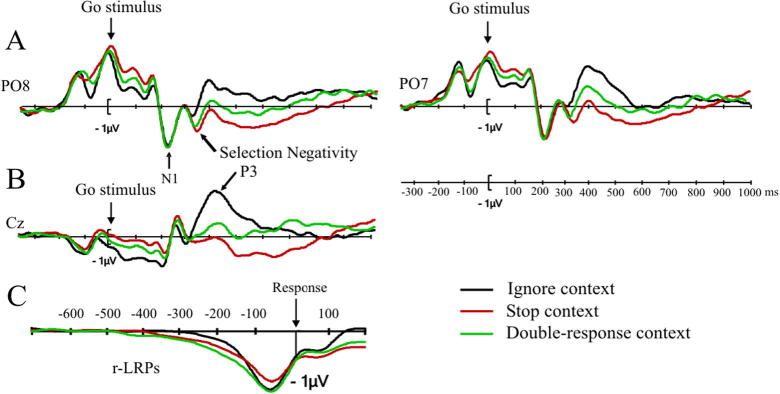
Experiment 2, post go stimulus waveforms for all contexts: A in electrodes PO8 (left) and PO7 (right), B in Cz, C response-locked LRPs.

As in Experiment 1 we examined the early perceptual components N1 and SN (see Fig.5A), as well as the P3 (Fig.5B), and the response-locked LRPs (Fig.5C). As mentioned above, we analyzed the ERPs for no-signal trials only.

##### 4.2.2.1 N1

N1 amplitudes were largest in the stop context (−7.6 μV), followed by the double-response context (−7.1 μV) and the ignore context (−6.3 μV). All differences were significant: stop versus ignore, *t*(29) = −4.48, *p* < .001, *d_av_* = 0.37, double-response versus ignore, *t*(29) = −3.0, *p* < .05, *d_av_* = 0.23, and double-response versus stop, *t*(29) = −2.71, *p* < .05, *d_av_* = 0.14. These findings are consistent with the results of Experiment 1 (although it should be noted that the numerical differences between the double-response and the other contexts were not significant in Experiment 1). Combined, these results suggest the perceptual analysis of the color stimulus is influenced by proactive control in the stop context, and to a smaller extent, in the double-response context. Note that an inspection of electrodes PO7 and PO8 also revealed some numerical differences between contexts preceding the onset of the go stimulus (during the cue to go stimulus interval). Since we had no predictions about these effects, we present the exploratory analysis of this effect in Appendix F.

##### 4.2.2.2 Selection Negativity

As for the N1, the SN amplitude was most negative in the stop context (−3.06 μV), followed by the double-response context (−2.88 μV) and the ignore context (−1.91 μV). The difference between the ignore context and the stop context was significant, *t*(29) = −6.0, *p* < .001, *d_av_* = 0.66, as was the difference between the ignore and double-response contexts, *t*(29) = −4.77, *p* < .001, *d_av_* = 0.55. The difference between the stop and double-response contexts was not significant, *t*(29) = −1.0, *p* = .33, *d_av_* = .1. The numerical differences are consistent with Experiment 1, but there the difference between the double-response and ignore context failed to reach significance after correction for multiple comparisons. We propose that the SN effects reflect differences in the feature analysis of the go stimulus: the analysis is more thorough to facilitate detection of a possible feature change (the signal) in the contexts in which subjects may have to change their actions. In other words, the SN is modulated by proactive adjustments of attentional settings to enhance detection of the (visual) signal.

##### 4.2.2.3 P3

Consistent with Experiment 1, we found a large (and early) P3 peak in the ignore context, a smaller P3 in the double-response context, and the smallest P3 in the stop context (Fig.5B, for an analysis with region as a factor see Appendix D). The difference between the ignore context and the stop and double-response contexts were significant, *t*(29) = 5.05, *p* < .001, *d_av_* = 0.884, and *t*(29) = 3.37, *p* < .01, *d_av_* = 0.536, respectively. This confirms that the response decision is easiest in the ignore context. While the P3 difference between the double-response and stop context failed to reach significance in Experiment 1, it was reliable in Experiment 2, *t*(29) = 3.65, *p* < .01, *d_av_* = 0.39. This is presumably due to a better signal to noise ratio resulting from the larger number of segments in the averages of Experiment 2. The difference between the double-response and stop contexts is consistent with the idea that response-thresholds are increased in stop-signal contexts but not (or to a lesser extent) in the double-response context (Verbruggen & Logan, 2009a); consequently, selecting the response takes longer in stop contexts than in double-response contexts.

##### 4.2.2.4 Lateralised Readiness Potential

The r-LRP (Fig.5C) reached 30% of peak amplitude at −226 ms in the double-response context, at −223 ms in the stop context, and at −155 ms in the ignore context. The onset latencies estimated by fitting the bilinear function were −484 ms for the double-response context, −402 ms for the stop context, and −318 ms for the ignore context. Some of these differences were significant for the 30% method (see Appendix E for details), but none was for the bilinear method (because of large inter-individual variability in some conditions). This means that the two r-LRP onset estimation methods did not converge for any of the contrasts, thus precluding firm conclusions from the above numerical differences. Peak latencies were −56 ms in the stop context, −58 ms in the double-response context and −60 ms in the ignore context. None of these differences were significant (all *p*’s > 0.5). Peak amplitudes were numerically smaller in the stop context than in the ignore context and the double-response context but these differences did not survive correction for multiple comparisons (corrected *p*’s: *p* = .1 and *p* = .06, respectively). The difference between the double-response and the ignore context was also not significant (*p* = .7).

## 5 Experiment 3

In Experiment 2, we found that the N1, the Selection Negativity and P3 were modulated in stop- and double-response contexts. Similar (numerical) differences were observed in Experiment 1. Combined, these experiments indicate that attentional selection and response selection in the go task are influenced when subjects prepare for a future act of action control. The difference in r-LRP onset between the double-response and ignore contexts in Experiment 1, though present numerically, was not conclusively confirmed.

In Experiment 3, we sought to provide additional evidence that the modulations of the N1 and the SN are indeed due to increased monitoring for the stop signal. To achieve the latter we decided to change the modality of the signal from visual to auditory. If the modulations of the N1 and SN reflect increased monitoring for a visual signal we should not find a modulation of these components in the current experiment; we tested this null hypothesis using Bayesian statistics.

### 5.1 Method

#### 5.1.1 Subjects, stimuli, procedure, and analyses

Thirty right-handed adults (20 female) with an average age of 22 (ranging from 18 to 46) were paid £12 to take part in this study. The paradigm was identical to the one in Experiment 2, with one exception: the visual signal was replaced by an auditory signal, a 500 Hz tone played over loudspeakers placed to the left and right of the screen. The EEG set-up and data processing procedures were identical to those in Experiment 2.

### 5.2 Results and discussion

#### 5.2.1 Behavioral results

Response times for no-signal trials in stop context (*M* = 673 ms, *sd* = 118 ms) were significantly longer than RTs in the ignore context (*M* = 456 ms, *sd* = 69 ms), *t*(29) = 13.2, *p* < 0.001, *d_av_* = 2.32, and RTs in the double-response context (*M* = 493 ms, *sd* = 73 ms), *t*(29) = 10.6, *p* < 0.001, *d_av_* = 1.89. The RT difference between the double-response and ignore contexts was also significant, *t*(29) = 6.4, *p* < 0.001, *d_av_* = 0.52.

The percentage correct go responses was higher in the stop context (*M* = 98.8%, *sd* = 1.7%) than in the ignore context (*M* = 96.9%, *sd* = 2.3%), *t*(29) = −5.12, *p* < .001, *d_av_* = 0.967, and in the double-response context (*M* = 98.2%, *sd* = 1.1%), *t*(29) = −2.91, *p* < .01, *d_av_* = 0.449. The difference between the ignore context and the double-response context was also significant, *t*(29) = −3.49, *p* < .01, *d_av_* = 0.753. As in Experiment 2, the number of missed responses was too low to warrant inferential statistics: stop context (*M* = 0.4%, *sd* = 0.7%), ignore context (*M* = 0.1%, *sd* = 0.3%), double-response context (*M* = 0.1%, *sd* = 0.3%).

#### 5.2.2 ERPs

Again, we analyzed the ERPs for no-signal trials only. The ERP averages for each context and subject contained about 150 trials.

##### 5.2.2.1 N1

**Fig. 6:**
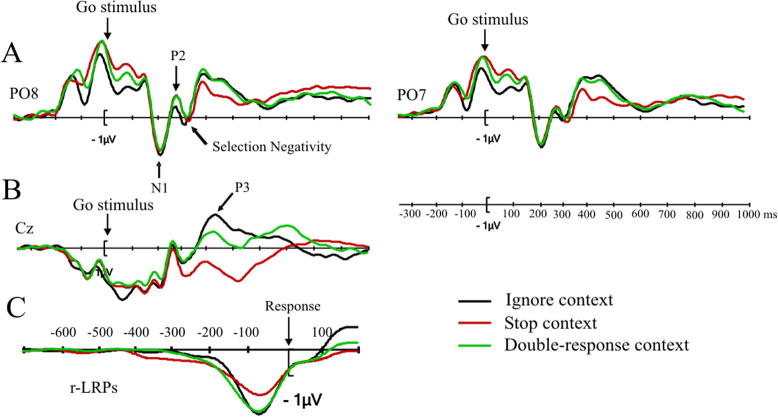
Experiment 3, post go stimulus waveforms for all contexts: A in electrodes PO8 (left) and PO7 (right), B in Cz, C response-locked LRPs.

Mean N1 amplitudes were −7.4 μV in the stop context, −7.5 μV in the double-response context, and −7.2 μV in the ignore context (Fig.6A). These differences between contexts were not significant (*p*’s > .3, uncorrected). To confirm the absence of the effect of context on the N1 amplitudes in Experiment 3, we calculated a Bayes factor, which compares two hypotheses: the hypothesis that the stop and double-response contexts modulate the amplitude of the N1 (compared to the ignore context) and the null hypothesis (i.e., no difference between contexts). Bayes factors vary between 0 and infinity with values of less than .33 indicating substantial support for the null hypothesis and values greater than 3 indicating substantial support for the alternative. We used Zoltan Dienes’ online calculator (http://www.lifesci.sussex.ac.uk/home/Zoltan_Dienes/inference/Bayes.htm) assuming a normal distribution with a mean of −1.07 μV (the numerical difference between the average N1 amplitude in the stop and double-response contexts and the N1 amplitude in the ignore context in Experiment 2), and a standard deviation that is half of the mean. The resulting Bayes factor was 0.32, indicating that the data provided substantial support for the null hypothesis. Thus, when a potential signal was presented in the auditory domain, the early perceptual analysis of the visual go stimulus did not differ between the dual-task and ignore contexts. This is in marked contrast to the results from Experiment 1, where there were significant differences in N1 amplitudes between the stop context and the ignore context, and Experiment 2, where there were significant differences between all three contexts.

##### 5.2.2.2 Selection Negativity

The amplitude of the SN was also numerically comparable in the three contexts (Fig.6A) and none of the contrasts approached significance (all *p*’s > .3, uncorrected). To confirm the null hypothesis, we calculated a Bayes factor comparing the hypotheses that stop and double-response contexts modulate the amplitude of the SN and the null (i.e., no difference between contexts). We assumed a normal distribution with a mean of −1.07 μV (the numerical difference between the average SN amplitude in the stop and double-response contexts and the amplitude in the ignore context in Experiment 2), and a standard deviation that is half of the mean. The resulting Bayes factor was 0.23, indicating that the data provided substantial support for the null hypothesis. In other words, the stop and double-response contexts did not modulate the amplitude of the SN when the double-response and stop signals were auditory.

##### 5.2.2.3 P3

The P3 amplitude was larger in the ignore context than in the stop context, *t*(29) = 5.54, *p* < .001, *d_av_* = 0.983 (Fig. 6B). The difference between the double-response and the stop contexts was also significant, *t*(29) = 6.1, *p* < .001, *d_av_* = 0.817, whereas the double-response vs. ignore difference was not, *t*(29) = 1.8, *p* = .5, *d_av_* = 0.220. For an analysis with scalp region as a factor, see Appendix D. In all the regions, amplitudes were less positive in the stop context than in the ignore- and double-response contexts. In sum, as in Experiment 2, the amplitude of P3 was smallest in the stop context and largest in the ignore context.

##### 5.2.2.4 Lateralised Readiness Potential

Response locked LRPs are shown in Fig. 6C. The r-LRP reached 30% of its amplitude at −241 ms in the stop context, at −176 ms in the double-response context and at −143 ms in the ignore context. Bilinear function fitting determined the r-LRP onset to be at −424 ms for the stop context, at −336 ms for the double-response context and at −296 ms for the ignore context. Although for each method some of the differences between conditions were statistically significant (for the 30% method it was the difference between the double-response and the ignore contexts; for the bilinear fitting it was the stop vs. ignore difference; see Appendix E), the two procedures did not converge for any contrast, thus precluding firm conclusions regarding differences between context in r-LRP onset. R-LRP peak amplitudes were smaller in the stop context (−1.9 μV) than the ignore context (−2.7 μV), *t*(29) = −4.45, *p* < .001, and the double-response context (−2.5 μV), *t*(29) = −3.04, *p* < .05. The difference between the double-response and the ignore context was not significant (*p* = 0.9). Peak latencies were −66 ms in the stop context, −70 ms in the double-response context and −68 ms in the ignore context. As in the other two experiments none of these differences were significant (all *p*’s > 0.6).

In Experiment 3, r-LRP peak amplitudes were smaller for the stop context compared to the ignore- and double-response contexts; similar numerical differences were also observed in Experiment 2, although there they failed to reach significance after correction. Combined, these findings could indicate that motor activity was reduced when preparing to respond in the stop context (Cai et al., 2011).

## 6 General discussion

Recent work on proactive inhibitory control has focused primarily on response-related processes (e.g. Aron, 2011, Lo et al., 2009, Stuphorn and Emeric, 2012, Verbruggen and Logan, 2009a), and neglected the role of attention. Furthermore, previous studies did not directly examine how proactive inhibitory control is related to other forms of proactive control and dynamic task-set reconfiguration. The present experiments measured ERPs to investigate whether people proactively adjust both attentional and response settings when they expect a stop signal in the environment (Study Aim 1). Second, we included a condition in which subjects had to execute a second response when a signal occurred to examine the specificity of the control adjustments (Study Aim 2). Third, to examine whether adjusting parameters proactively in anticipation of a stop signal is analogous to adjusting task-set parameters during a task switch, Experiment 1 measured the previously documented neurophysiological ‘signature’ of preparing to switch tasks during preparation for a stop signal (Study Aim 3). Combined, the results of Experiments 1–3 provide clear answers to the three main questions. We will now review the main findings and discuss their theoretical implications.

### 6.1 A biased competition account of proactive inhibitory control

#### 6.1.1 Proactive modulation of attentional and response settings in stop contexts

Recent studies of response inhibition indicate that people proactively adjust response settings in anticipation of future acts of control. However, finding a balance between ignoring irrelevant stimuli in the environment and monitoring for potentially relevant control signals could also be an integral part of proactive inhibitory control, and more generally, flexible and goal-directed behavior (Goschke & Dreisbach, 2008).

In Experiments 1 and 2, amplitudes of the N1 and the Selection Negativity were larger in the stop context than in the ignore context. The occipital N1 reflects the detection of a visual stimulus and the Selection Negativity has been related to feature selection in a visual stimulus. Therefore, Experiments 1 and 2 indicate that the processing of the go stimulus is influenced by anticipation of a stop signal. The ‘biased competition’ account of visual attention addresses the issue of how the perceptual system deals with limited processing capacity in the face of the large amount of visual information provided by the environment (e.g., Desimone and Duncan, 1995, Duncan, 2006, Kastner and Ungerleider, 2000). This theory assumes that there is competition between the different sources of information. Attention biases specific features, objects or representations to resolve this competition. We propose that stop-signal detection can be biased by increasing the baseline activity in sensory neurons that code for specific stop-signal related features; consequently, those features are more likely to win the competition, and hence, stopping is more likely to succeed. The results of Experiment 3 further support the interpretation of the N1 and Selection Negativity as markers of monitoring for a *visual* signal because they were not modulated by context when the signal was auditory. In other words, when the stop signal is a loud auditory tone, proactive inhibitory control seems to have little or no influence on the processing of the visual go stimulus, because there is less competition compared to when both the go stimulus and the stop signal are both in the visual modality (see e.g. Logan et al., 2014). Note that this does not imply that subjects do not have to monitor for auditory signals; our ERP results only indicate that the increased monitoring demands do not interfere with the processing of the visual go stimulus.

The analyses of the P3 and r-LRP showed that response settings were also modulated in the stop context. P3 amplitudes were larger in the ignore context, in which subjects had to execute a single go response on all trials, than in the stop context. Our previous work indicates that proactive adjustments can influence the parameters of response selection (Verbruggen & Logan, 2009a). For example, we found that response thresholds were higher in stop contexts than ignore contexts, explaining the longer RTs in the stop context (Logan et al., 2014, Verbruggen and Logan, 2009a). Although we did not fit the diffusion model to the current data, the behavioral results suggest that subjects adjusted response selection parameters in the stop context: RTs increased significantly, and go accuracy also tended to be higher in the stop context than in the ignore context. The differences in P3 could in part reflect such strategic adjustments.

The reduced r-LRP amplitude in the stop context of Experiment 3 is likely due to a combination of at least two response-execution related factors. First, it might be a reflection of reduced activation of the motor cortex when subjects anticipate a stop signal (Cai et al., 2011, Lo et al., 2009, Wiecki and Frank, 2013, Wong-Lin et al., 2010). For example, Cai et al. (2011) directly measured corticomotor excitability on go trials in a stop context (cue signaling ‘maybe stop left/right’) by means of motor evoked potentials. They found that motor evoked potentials were significantly smaller for the finger that potentially needed to be stopped compared to when the finger was at rest. Secondly, increased variability in planning the motor response when there might be the need to stop might contribute to smaller r-LRP amplitudes in the stop context (although it should be noted that the r-LRP onset comparisons were inconclusive).

The present study and previous empirical work suggests that control adjustments before a stop signal appears are critical for successful response inhibition. Proactive inhibitory control could even lead to a ‘prepared inhibition reflex’ (Verbruggen et al., 2014, Verbruggen and Logan, 2015; for a review of the wider ‘prepared reflex’ literature, see Meiran, Cole, & Braver, 2012). When attention is biased and the stop response is prepared, response inhibition on stop-signal trials may not require much control anymore. Consistent with this idea, response inhibition can be triggered by task-irrelevant primes (e.g. van Gaal et al., 2009, Verbruggen and Logan, 2009c), but these priming effects are only observed in contexts in which subjects are instructed to stop occasionally (Chiu and Aron, 2014, Verbruggen and Logan, 2009c). These findings are consistent with the prepared reflex idea: once subjects have proactively adjusted attentional and response settings, the ‘stop response’ can be activated easily by information in the environment. Note that the ‘prepared reflex idea’ might also explain why going and stopping do not share (much) capacity in standard stop-signal tasks (Verbruggen & Logan, 2015).

#### 6.1.2 Domain-general vs. domain specific control adjustments

We included the double-response context to examine the generality of proactive inhibitory control adjustments (i.e. strategic adjustments made in anticipation of a stop signal). In Experiment 2, we found that the N1 and the Selection Negativity were larger in the double-response context than in the ignore context. This indicates that subjects also monitored for double-response signals. However, the absence of significant differences between double-response and ignore contexts in Experiment 1, and the differences between stop and double-response contexts in Experiment 2 suggest that the overall monitoring demands must have been lower than in the stop context.

The analyses of the P3 also revealed differences between the double-response context and the ignore context, although they were smaller than the differences between the ignore- and stop contexts. As discussed in Experiment 1, P3 differences could be due to increased uncertainty, divided attention, and increased response selection demands. In Verbruggen and Logan (2009a), we did not find modulations of the decision-making parameters in a double-response context (but a comparison of the behavioral results indicates that the double-response condition in the current study was more difficult than the double-response condition in Verbruggen & Logan, 2009a). Thus, our results indicate that subjects also adjust response settings in the primary task when they anticipate double-response signals to occur.

Finally, the r-LRP onset results of Experiment 1 show a delay in action programming in the double-response context compared with the ignore context. This is consistent with results of Smulders, Kok, Kenemans, and Bashore (1995), who found that response complexity (which was manipulated by having subjects press different sequences of keys with three different fingers) increased the interval between r-LRP onset and the execution of the first key press. Our observation of modulations by context of a component that arises after the response decision suggests that even the last stage of action programming, just before the response is executed, can also be influenced by competition and interference from the response required by the signal.

In sum, even though there were quantitative differences between the stop and double-response context at some stages, both were distinctly different from the ignore context. This suggests that proactive ‘inhibitory’ control involves making adjustments that are also made in other (non-inhibitory) task contexts. This is consistent with the idea that proactive inhibitory control involves biasing the competition between sources of information and action options (see above). It is also consistent with the idea that the effects of various acts of control are generally the same: they all change the parameters of underlying selection processes (Logan et al., 2014). In other words, the distinction between different acts of proactive control (including proactive inhibitory control and task switching) may be primarily in *which* parameters are changed, but not in *how* they are changed.

#### 6.1.3 The posterior positivity as a marker of proactive adjustments?

Experiment 1 revealed a substantial (∼30% or more of the ERP amplitude) and protracted (>400 ms) positive-polarity ERP deflection elicited over the posterior scalp by a switch to the stop context relative to a repetition of this context. This brain potential has all the features of the ‘positivity’ elicited in previous studies by preparation for a task switch; the latter is widely believed to reflect endogenous task-set reconfiguration (e.g., Karayanidis et al., 2003, Karayanidis et al., 2011, Lavric et al., 2008; but see e.g., Kang et al., 2014 who argued that it reflects bottom-up retrieval of cue–stimulus associations). The presence of this component in Experiment 1 suggests that there is considerable overlap between preparation for inhibitory control and the control processes engaged when preparing for a switch of tasks. We propose that in both cases, preparation involves adjusting the parameters of attention- and response selection components of a task-set to support flexible behavior.

One aspect of the switch-related positivity documented here may help gain further insight not only into proactive control in the stop-signal paradigm, but proactive control of task-sets in general. In task-switching, the positivity is found when a switch involves changing attentional and response settings, but it is also found when only one of these components must change. Moreover, it does not seem to matter which task-set component switches, as shown by Rushworth et al., 2002, Rushworth et al., 2005 and Hsieh and Wu (2011), who found that both switching between stimulus dimensions (without changing the response rules) and switching response rules (without changing the relevant stimulus dimension) resulted in a posterior positivity. Recent unpublished work in our laboratory indicates that the positivity is also elicited when bilinguals are asked to prepare to switch the language for production (in the absence of a change of task or stimulus dimension), and when subjects prepare to change the response threshold in a paradigm that requires trial-to-trial switching in the speed-accuracy criterion (in a constant task without a change in stimulus dimensions or response rules).

Against this background of apparent non-specificity of the positivity, it is intriguing that in our Experiment 1 the positivity was robust for switches to the stop context, yet it seemed absent for switches to the ignore context (in the double-response context, we observed a numerical difference between context switches and context repeats but it was not statistically significant). The present pattern of results shows that having to add an attentional component (to monitor for the signal) or an S-R rule (to suppress the response in the primary task if a signal is presented) to the task-set does not yield the same electrophysiological profile as ‘removing’ these task-set components. In particular, the activation or retrieval of these extra components of the task-set (in the stop context) elicits a positivity 400–800 ms after the cue presentation, whereas the removal of the need to activate or retrieve them (in the ignore context) does not result in a discernable late positivity. This suggests that advance task-set reconfiguration as measured by the (late) posterior positivity is predominantly the activation of (components of) the new/relevant task-set, rather (or more so) than the suppression of the no longer relevant task-set. This idea is consistent with findings of Karayanidis and colleagues (e.g. Karayanidis et al., 2009, Nicholson et al., 2006), who found that the switch-related positivity in their studies consisted of an early and a late subcomponent or phase. The early subcomponent was elicited by both switch-to cues (subjects were told to which task they had to switch; these cues are similar to the stop cues used in the present study) and switch-away cues (subjects were informed that the task would switch but the to-be-performed task was only specified upon target onset). The late subcomponent was only observed for switch-to-cues. Based on this finding, Karayanidis and colleagues argued that the early component reflects inhibition of the no-longer relevant task (which can occur for both switch-to and switch-away cues), whereas the late component reflects advance task-set reconfiguration (which can occur only for switch-to cues). In the present study, we focused on the late component or phase. Thus, the presence of a posterior positivity for the stop context but the absence for the ignore context seems consistent with Karayanidis et al.’s finding that the late component is observed for switch-to cues but not for switch-away cues.

Finally, most proactive inhibitory control studies contrast short blocks of trials in which subjects have to stop with blocks in which they can respond on all trials. Furthermore, some have argued that response settings can only be adjusted at the beginning of a block (see above). Our ERP findings indicate that subjects can proactively adjust attentional and response settings on a trial-by-trial basis, replicating our previous behavioral findings (Verbruggen & Logan, 2009a). Thus, proactive inhibitory control is highly dynamic.

### 6.2 Benefits and costs of proactive control

Behavioral and computational work indicates that proactive control could be beneficial. For example, Lo et al. (2009) presented a neural circuit model of proactive inhibitory control in a countermanding paradigm, in which subjects have to suppress eye movements. Suppression of eye movements on stop-signal trials involves the activation of fixation neurons (STOP unit), which inhibit movement neurons (GO unit; Boucher et al., 2007). Lo et al. observed high tonic activation of fixation neurons before the onset of a stop signal, and argued that this was modulated by a top-down proactive control signal. Importantly, their model showed that whether or not an eye movement was stopped depended to a large extent on pre-signal activation of fixation neurons (and consequently, the proactive suppression of motor neurons). Thus, proactive control could be highly beneficial. However, it can also come with certain costs. Proactive control cannot always be maintained over long periods of time because of the high metabolic cost of actively maintaining relevant information (Braver et al., 2007) and because of interference caused by other ongoing tasks (e.g. Blackwell & Munakata, 2014). Proactive control is also in opposition to a fast, efficient automatization of processes because adhering to contextual goals can override fast default processing or result in insensitivity to unexpected environmental changes. Finally, proactive control might compete for resources with other tasks (Braver et al., 2007). For example, our recent study showed that subjects were more distracted when they monitored the periphery for stop signals (Verbruggen, Stevens, et al., 2014). In sum, proactive adjustments seem an inherent part of response inhibition and executive control, but it requires finding an optimal balance to be effective.

## 7 Conclusions

Our results show that proactive inhibitory control works by biasing or altering the settings of lower-level systems that are involved in selecting the relevant stimulus, selecting the appropriate action, and executing it. Furthermore, we have demonstrated that similar adjustments are made for various forms of control. The results of Experiment 1 also suggest considerable overlap between preparatory control in task-switching studies and proactive action control. Our biased competition account of proactive inhibitory control is consistent with work in the visual attention domain and the dual mechanisms framework of Braver and colleagues (e.g. Braver, 2012), which deals primarily with proactive control in interference and working-memory tasks. Thus, the present study pulls ‘proactive inhibitory control’ out of the response inhibition niche and grounds it more firmly in the wider attention and action control literature.

## Figures and Tables

**Table A1:** Mean RTs in ms for first and second responses on double-response signal trials as a function of SOA for Experiments 1–3.

	Short SOA	Medium SOA	Long SOA
*Experiment 1*			
RT 1	778	797	785
RT 2	963	875	836

*Experiment 2*			
RT 1	581	602	588
RT 2	612	544	553

*Experiment 3*			
RT 1	502	521	490
RT 2	638	548	507

**Fig. A2:**
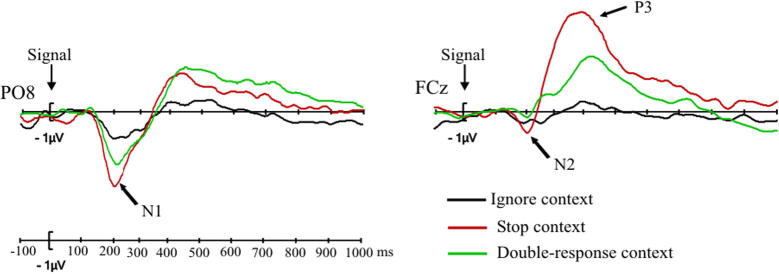
Experiment 2: waveforms of signal trials in electrodes PO8 and FCz.

**Fig. A3:**
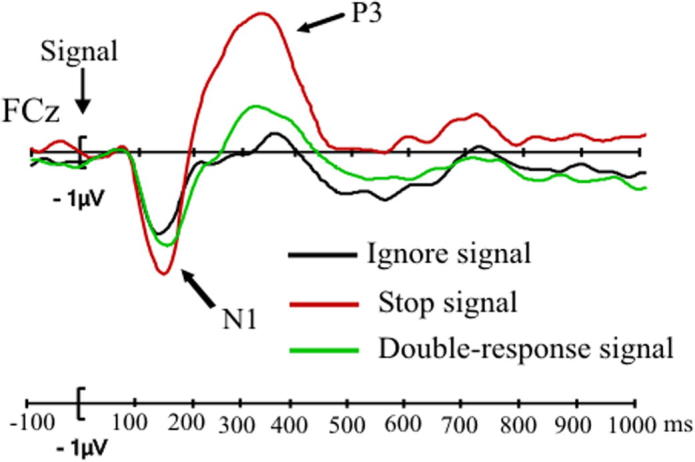
Experiment 3: waveforms for signal trials in electrode FCz.

**Table D1:** Statistics of amplitude differences between contexts for each region in the P3 range (corrected *p*-values are shown) in Experiment 1.

Region	Contrasts	*t*	*p*	*d_av_*
FrPm	Stop vs. ignore	−3.81	<.01	0.71
Double-response vs. ignore	−4.37	<.01	0.59
Stop vs. double-response	−0.93	0.4	0.13

Pm	Stop vs. ignore	−3.9	<.01	0.57
Double-response vs. ignore	−4.24	<.01	0.49
Stop vs. double-response	−1.34	0.2	0.15

Om	Stop vs. ignore	−3.06	<.05	0.42
Double-response vs. ignore	−2.58	0.06	0.24
Stop vs. double-response	−1.81	0.08	0.17

**Table D2:** Statistics of amplitude differences between contexts for each region in the P3 range (corrected *p*-values are shown) for Experiment 2.

Region	Contrasts	*t*	*p*	*d_av_*
FrPm	Stop vs. ignore	−5.45	<.001	0.82
Double-response vs. ignore	−4.14	<.01	0.54
Stop vs. double-response	3.43	<.05	0.31

Pm	Stop vs. ignore	−4.56	<.001	0.81
Double-response vs. ignore	−3.08	<.05	0.48
Stop vs. double-response	−3.41	<.01	0.39

Om	Stop vs. ignore	−4.13	<.01	0.70
Double-response vs. ignore	−2.36	<.05	0.40
Stop vs. double-response	−3.11	<.01	0.32

**Table D3:** Statistics of amplitude differences between contexts for each region in the P3 range (corrected *p*-values are shown) for Experiment 3.

Region	Contrasts	*t*	*p*	*d_av_*
FrPm	Stop vs. ignore	−6.63	<.001	1.24
Double-response vs. ignore	−2.51	0.054	0.41
Stop vs. double-response	−6.05	<.001	0.93

Pm	Stop vs. ignore	−5.03	<.001	0.87
Double-response vs. ignore	−1.38	0.18	0.13
Stop vs. double-response	−5.6	<.001	0.77

Om	Stop vs. ignore	−3.42	<.01	0.51
Double-response vs. ignore	−0.45	0.65	0.04
Stop vs. double-response	−4.53	<.001	0.47

**Table E1:** R-LRP onset estimations using the 30% of peak amplitude criterion and bilinear function fitting for all three experiments.

Contrast	30% criterion mean diff, *t*-statistics	Bilinear function fitting mean diff; *t*-statistics
*Experiment 1*		
Stop vs. ignore	18 ms, *t*(31) = −0.77, *p* = .4	116 ms, *t*(31) = −2.88, *p* < .05
Double-resp. vs. ignore	51 ms, *t*(31) = −3.06, *p* < .05	188 ms, *t*(31) = −5.18, *p* < .001
Stop vs. double-resp.	33 ms, *t*(31) = 1.67, *p* = .2	72 ms, *t*(31) = −2.58, *p* < .05

*Experiment 2*		
Stop vs. ignore	68 ms, *t*(29) = −2.32, *p* = .054	84 ms, *t*(29) = −0.84, *p* = .4
Double-resp. vs. ignore	71 ms, *t*(29) = −2.77, *p* < .05	166 ms, *t*(29) = −1.71, *p* = .09
Stop vs. double-resp.	3 ms, *t*(29) = 0.13, *p* = .9	82 ms, *t*(29) = −1.54, *p* = .1

*Experiment 3*		
Stop vs. ignore	98 ms, *t*(29) = −2.21, *p* = .07	128 ms, *t*(29) = −4.4, *p* < .001
Double-resp. vs. ignore	176 ms, *t*(29) = −15, *p* < .05	40 ms, *t*(29) = −077, *p* = .4
Stop vs. double-resp.	65 ms, *t*(29) = −1.59, *p* = .1	88 ms, *t*(29) = −1.62, *p* = .1

**Table 1:** Mean (*sd* in parentheses) RTs and switch costs (*se* in parentheses) for no-signal trials in all conditions (in ms).

	Long CSI			Short CSI		
Switch	Repeat	Switch cost	Switch	Repeat	Switch cost
Ignore	604 (115)	593 (118)	11 (6)	685 (101)	692 (151)	−7 (15)
Double	684 (134)	675 (136)	9 (4)	781 (131)	764 (140)	17 (10)
Stop	755 (124)	773 (128)	−17 (7)	812 (124)	815 (125)	−3 (12)

**Table 2:** Mean error rates (*sd* in parentheses) and error switch costs (*se* in parentheses) for no-signal trials in all conditions (in %).

	Long CSI			Short CSI		
Switch	Repeat	Switch cost	Switch	Repeat	Switch cost
Ignore	1.9 (1.9)	2.3 (2.4)	−0.4 (0.5)	3.6 (3.4)	4.3 (5.1)	−0.7 (1)
Double	1.9 (1.6)	1.7 (1.8)	0.2 (0.4)	3.9 (4.6)	2.8 (3.6)	1.1 (1.1)
Stop	1.5 (1.7)	1.1 (1.3)	0.4 (0.4)	2.0 (2.6)	1.6 (3.2)	0.4 (0.7)

**Table 3:** Overview of repeated measures ANOVAs on RTs and error rates.

	*df* 1	*df* 2	*F*	*p*	Partial *η*^2^
**RTs**					
CSI	1	31	119.1	<.001	0.793
Context	2	62	43.7	<.001	0.585
Switch	1	31	0.21	0.65	0.007
Context × CSI	2	62	8.23	<.01	0.120
Context × switch	2	62	2.69	0.95	0.080
CSI × switch	1	31	0.54	0.81	0.002
Context × CSI × switch	2	62	1.49	0.23	0.046

*Ignore context only*					
CSI	1	31	83.45	<.001	0.729
Switch	1	31	0.56	0.81	0.002
CSI × switch	1	31	1.20	0.23	0.037

*Double-response context only*					
CSI	1	31	67.65	<.001	0.686
Switch	1	31	7.85	<.01	0.202
CSI × switch	1	31	0.58	0.45	0.018

*Stop context only*					
CSI	1	31	37.87	<.001	0.550
Switch	1	31	2.12	0.16	0.064
CSI × switch	1	31	1.16	0.29	0.036

**Errors**					
CSI	1	31	22.61	<.001	0.422
Context	2	62	10.20	<.001	0.248
Switch	1	31	0.20	0.66	0.006
Context × CSI	2	62	2.31	0.1	0.069
Context × switch	2	62	1.6	0.21	0.049
CSI × switch	1	31	0.82	0.77	0.003
Context × CSI × switch	2	62	0.41	0.67	0.013

*Ignore context only*					
CSI	1	31	9.29	<.01	0.231
Switch	1	31	0.81	0.38	0.025
CSI × switch	1	31	0.12	0.74	0.004

*Double-response context only*					
CSI	1	31	12.98	<.01	0.295
Switch	1	31	1.25	0.27	0.039
CSI × switch	1	31	0.60	0.45	0.019

*Stop context only*					
CSI	1	31	1.81	0.19	0.055
Switch	1	31	0.75	0.39	0.024
CSI × switch	1	31	0.001	0.98	<.001

## Appendix A Analysis of signal trials

### A.1 Behavioral data

#### A.1.1 Experiment 1

On ignore signal trials, subjects responded slower (82 ms) to the go stimulus than on the no-signal trials, *t*(31) = 5.65, *p* < .001, *d_av_* = .58. This ignore-signal slowing was much more pronounced than in our previous studies in which contexts were presented in mini-blocks of 4–8 trials in a predictable order (e.g., Verbruggen and Logan, 2009a, Verbruggen et al., 2010). Thus, the substantial ignore slowing in the present study is probably due to increased context conflict and/or context inertia (similar to task-set conflict/inertia in task switching) caused by the frequent switching between task contexts, rather than by sensory distraction.

On double-response signal trials, RTs of the first response were 62 ms slower than on no-signal trials, *t*(31) = 5.02, *p* < .001, *d_av_* = .44. Again, this RT increase is probably due to context conflict and/or context inertia. Table A1 shows the latencies of the first and second response as a function of SOA. RTs of the second response differed reliably as a function of SOA, *F*(2, 62) = 55.55, *p* < .001, partial *η*^2^ = 0.629, whereas RT of the first response was not reliably influenced by SOA, *F*(2, 62) = 0.7, *p* = .5, partial *η*^2^ = 0.022.

The inhibition functions depicting the relation between *p*(respond|signal) and the stop signal delay (SSD) is shown in Fig. A1. The independent horse-race model (Logan & Cowan, 1984) assumes that the SSD will influence the relative finishing time of the stop process: when SSD increases, the stop process will start later and therefore, finish later compared to the go process; consequently, response inhibition will succeed less often. The inhibition function for Experiment 1 is consistent with the predictions of the independent race model, suggesting that a stop process was required to withhold the response when a signal occurred. We did not estimate stop-signal latencies because the overall probability of responding on stop-signal trials was very low (21% on average, for some people as low as 6–7%),^5^ which would have resulted in unreliable SSRT estimates (Band, van der Molen, & Logan, 2003).

#### A.1.2 Experiment 2

In the ignore context, RTs were longer on signal trials (*M* = 475 ms, *sd* = 128 ms) than on no-signal trials, but this difference was not reliable, *p* = .25. Again, this suggests that the large difference between no-signal trials and ignore-signal trials in Experiment 1 was due to some form of context inertia or rule conflict caused by the switching of contexts.

In the double-response context, RTs were slightly longer on signal trials (*M* = 588 ms, *sd* = 149 ms) than on no-signal trials. As in the ignore context, this difference was not significant, *p* = 0.4. Table A1 shows the latencies of the first and second response on double-response signal trials as a function of SOA. Latencies of second responses differed reliably as a function of SOA, *F*(2, 58) = 65.17, *p* < .001, partial *η*^2^ = .692, but latencies of first responses did not, *F*(2, 58) = 2.63, *p* = .095, partial *η*^2^ = .083.

As in Experiment 1, the inhibition function is consistent with the race model (Fig. A1). Again, probability of responding on a stop signal trial was too low (21%) to allow a valid estimation of SSRTs.

#### A.1.3 Experiment 3

In the ignore context, RTs were 13 ms longer on signal trials than on no-signal trials, *t*(29) = 2.64, *p* < .05, *d_av_* = 0.18. In the double-response context, first responses were also longer on signal trials (*M* = 505 ms, *sd* = 74 ms) than RTs on no-signal trials (*M* = 493 ms, *sd* = 73 ms), *t*(29) = 3.39, *p* < .01, *d_av_* = 0.16. Table A1 gives the latencies of the first and second response on double-response signal trials as a function of SOA. Latencies of second responses decreased significantly as a function of SOA, *F*(2, 58) = 162.41, *p* < .001, partial *η*^2^ = .848. Latencies of first responses also differed as a function of SOA, *F*(2, 58) = 15.42, *p* < .001, partial *η*^2^ = .347. Table A1 indicates that latencies were longest for the medium SOA.

As in the previous experiments, the probability of responding to a stop signal was too low (24%) to estimate SSRTs, but see Fig. A1 for the inhibition function.

#### A.1.4 Exploratory analysis of signal ERPs in Experiments 2 and 3

For signal trials the EEG was first cut into a long segment starting from 100 ms preceding the go stimulus, including the SOA, to 1000 ms following signal onset, and baseline corrected to the 100 ms preceding the go stimulus (to avoid stimulus-related activity contaminating the baseline). Then, a smaller segment was cut from −100 ms to 1000 ms time locked to the signal. We only chose trials from the longest SOA since ERPs for the short and medium SOA are contaminated with overlapping activity elicited by the preceding go stimulus. Furthermore, at short and medium SOAs, the refractoriness of ERPs could lead to a false assessment of signal trial amplitudes (Woodman, 2010). As described above, SOAs were calculated individually for each subject and each condition.

Average durations of the longest SOA were: 521 ms (ranging from 398 to 701 ms) in the stop context, 464 ms (ranging from 291 to 731 ms) in the double-response context, and 356 ms (ranging from 254 to 521) in the ignore context. Large signal-respond rates in the longest SOA in the stop condition resulted in very low trial numbers in seven subjects, who were therefore excluded for the signal analyses. Low trial numbers in the averages of the remaining subjects together with the exclusion of subjects made a statistical analysis of these waveforms questionable but we present signal trial data at the descriptive level (plots of grand-average ERP waveforms).

Waveforms time locked to the signal in electrodes PO8 and FCz for Experiments 2 and 3 are shown in Fig. A2, Fig. A3, respectively. In Experiment 2, the visual N1 amplitude differed substantially between contexts, suggesting a sensory gain due to increased attention for the stop signal and (albeit to a smaller extent) the double-response signal, compared with the ignore signal. Only the stop signal seemed to elicit an N2-like component. Amplitudes of the fronto-central P3 differed substantially between contexts: they were largest for the stop signal, medium for the double-response signal, and nearly absent for ignore signals.

In Experiment 3, the auditory N1 differed substantially between contexts, with largest amplitudes for stop signals compared with the double-response and ignore signals. Amplitudes for double-response signals were slightly larger than for ignore signals. We could not detect a stop-specific modulation of the N2 in this experiment, possibly due to the large magnitude of overlapping components (auditory N1 and following fronto-central P3). P3 amplitudes were largest in the stop condition, but were also increased for double-response signals compared with ignore signals.

## Appendix B Estimating the onset of the Lateralised Readiness Potential by fitting a bilinear function

Among procedures used for estimating the LRP onset, least-square fitting a bilinear function to use the inflection point as the onset estimate may have important advantages over other methods (e.g., Mordkoff & Gianaros, 2000). It computes an actual numerical estimate of the LRP onset, unlike the more widely used criterion-based method, which compares the latency at which the LRP in different conditions reaches a certain % of the maximal LRP amplitude, without providing an onset estimate per se. Another advantage over the criterion-based method is that the estimate based on bilinear fitting is independent of the maximal LRP amplitude, whereas the criterion-based measure is not. However, bilinear fitting has its limitations because its outcomes can be heavily influenced by the morphology of the EEG – the horizontal portion preceding the LRP and the extent to which the rising portion of the LRP deviates from linearity. We therefore sought convergence between these two procedures in estimating the response-locked LRP onset (see the Method section in the Main Text). Our use of the criterion-based procedure is presented in the Main Text. Here we outline how we used bilinear function fitting.

For each jack-knifing sub-average, we fitted a bilinear function, which consisted of a horizontal portion and a negatively rising portion. We allowed the total length of the fitted portion to vary between 50 time-points and 75 time-points (100 ms and 150 ms), whilst also allowing the length of each of two sub-segments (the horizontal and the rising portions) to vary with the constraint that each of them is at least 20 time-points (40 ms) long – we imposed this constraint to avoid fitting local noise (brief fluctuations in either sub-segment). The fitting started by determining, by visual inspection, an approximate LRP onset and providing the fitting algorithm an ‘initial starting point’ which was set 32 time-points earlier [half of the range of average length of the fitted segment: (50 + 75)/2] than the approximate LRP onset determined visually. The algorithm then fitted segments that started there, as well as 1–50 ms later or 1–50 ms earlier than the initial starting point, in increments of 2 ms (1 time-point). For each of these iterations:

– the horizontal portion of the bilinear was allowed to vary between −0.5 mV and 0.5 mV (this range exceeded any voltages in all the jackknifing sub-averages for the respective time-window) in increments of −0.05 mV;
– the slope of the rising portion was allowed to vary, such that the maximal slope corresponded to an end-of-segment voltage of −1 mV (more negative than in any of the jackknifing sub-averages) and the minimal slope corresponded to an end-of-segment voltage of −0.1 mV (substantially less negative than in any of the jackknifing sub-averages) in increments of 0.05 mV.

For each jackknifing sub-average, we applied standard least-square fitting and selected the starting point, length of the fitted bilinear function, amplitude of the horizontal portion and slope that resulted in the smallest sum-of-squared residuals scaled by (divided by) segment length in time-points (this scaling was necessary, because otherwise the shorter segments would always result in smaller residuals). The inflection point of the bilinear function from the iteration that resulted in the smallest residual was recorded as the r-LRP onset for the respective jackknifing sub-average.

## Appendix C Does switching influence the N1, SN, and P3 in Experiment 1?

We tested for effects of switching in the different contexts on the N1, SN and P3 components (the r-LRP calculation requires a further subdivision by left and right hand responses, which did not leave enough trials). The division of segments by switch-repeat (only one third repeat trials) resulted in much noisier waveforms and it was not possible to detect unambiguous peaks for the N1 and SN in a number of participants. We therefore used Miller et al.’s (1998) jackknifing procedure to detect peak amplitude differences in the N1 and SN. For the P3 analysis this was not necessary because here amplitudes were averaged between 300 and 500 ms to capture its broad peak. N1 amplitudes were measured as the P1-N1 difference and SN amplitudes as the P2-SN difference, averaged over electrodes PO7 and PO8. *T*-tests comparing switch and repeat showed no significant effects in any context for neither of the components (uncorrected *p*-values are shown): N1, stop context, *t*(31) = 0.84, *p* = .4; N1, double-response context, *t*(31) = 0.53, *p* = .6; N1, ignore context, *t*(31) = 0.69, *p* = .5; SN, stop context, *t*(31) = 0.12, *p* = .7; SN, double-response context, *t*(31) = −0.08, *p* = .9; SN, ignore context, *t*(31) = 0.99, *p* = .3. *T*-tests on P3 amplitudes averaged between 300 and 500 ms across three midline regions did not reveal any effects of switching either: stop context, *t*(31) = −0.7, *p* = .5; double-response context, *t*(31) = −1.58, *p* = .1; ignore context, *t*(31) = 0.06, *p* = .9.

## Appendix D A region analysis of the P3 in Experiments 1–3

We tested for differences between regions (P3a, P3b) with an ANOVA including the factors context and region. In Experiment 1, amplitudes in all the tested regions were modulated by context, *F*(2, 62) = 12.76, *p* < .001, partial *η*^2^ = .29. There was no reliable interaction with scalp region (*p* = 0.07, partial *η*^2^ = .087). In Experiment 2, all regions showed reliable differences between contexts, *F*(2, 58) = 18.23, *p* < .01, partial *η*^2^ = .386, but with some regional differences in the magnitude of the effect, *F*(4, 116) = 3.19, *p* < .05, partial *η*^2^ = .099. In Experiment 3, all regions showed reliable differences between contexts, *F*(2, 58) = 18.23, *p* < .01, partial *η*^2^ = .386, but with some regional differences in the magnitude of the effect, *F*(4, 116) = 3.19, *p* < .05, partial *η*^2^ = .099 (see Table D1, Table D2, Table D3).

## Appendix E Statistics for the r-LRP onset comparisons in Experiments 1–3

See Table E1.

## Appendix F An exploratory analysis of the posterior positivity in Experiments 2–3

Experiment 2 also revealed an unexpected ERP feature: visual inspection of the ERP waveforms suggests that amplitudes between contexts already differed quite considerably at the onset of the color stimulus (see Fig. 5). This could reflect a preparation-related positivity: Given that context was 100% predictable in this experiment, subjects could prepare for action control before the stimulus turned yellow or green. Similar to the preparation-related posterior positivity in Experiment 1, amplitudes were most positive in the stop context, slightly less positive in the double-response context, and least positive in the ignore context (see Fig.5A and B). An exploratory analysis of the amplitudes averaged between 0 and 100 ms following the go stimulus, with context, scalp region and laterality (Fig. 2) as within-subjects factors showed a reliable main effect of context, *F*(2, 58) = 22.19, *p* < .001 (uncorrected), partial *η*^2^ = .434. Subsequent tests revealed that differences between contexts were all reliable: stop vs. ignore: *F*(1, 29) = 52.99, *p* < .001, partial *η*^2^ = .646; stop vs. double-response, *F*(1, 29) = 9.86, *p* < 0.01, partial *η*^2^ = .254; and double-response vs. ignore: *F*(1, 29) = 10.66, *p* < .001, partial *η*^2^ = .269. The distribution of this component is similar to the distribution of the posterior switch positivity observed in Experiment 1, and might reflect some form of context-related preparation.

We also observed this posterior positivity in Experiment 3: Amplitudes already differed between contexts at and immediately following the color change of the stimulus (at 0–100 ms, see Fig. 6), *F*(2, 58) = 13.28, *p* < .01, partial *η*^2^ = .314. Amplitudes were more positive in the stop and double-response contexts than in the ignore context, *F*(1, 29) = 29.64, *p* < .001, partial *η*^2^ = .505, and *F*(1, 29) = 9.68, *p* < .01, partial *η*^2^ = .25, respectively. Amplitudes were more positive in the stop context than in the double-response context, but only in right lateralized, posterior regions of the scalp (main effect: *F*(1, 29) = 2.04, *p* = .16, partial *η*^2^ = .066; context by region interaction: *F*(3, 87) = 9.77, *p* < .001, partial *η*^2^ = .252; context by laterality: *F*(2, 58) = 3.44, *p* < .05, partial *η*^2^ = .106.

## Footnotes

1 The right inferior frontal junction was primarily involved in detecting the infrequent signals in the double-response and stop-signal contexts (attentional selection), whereas the right inferior frontal gyrus was primarily involved in selecting the double-response or the stop response (action selection). These findings indicate that the right ventrolateral prefrontal cortex is involved in both attentional and action selection in various action control tasks. This idea is further supported by recent neuro-imaging studies (e.g., Dodds et al., 2011, Erika-Florence et al., 2014, Hampshire et al., 2010, Levy and Wagner, 2011).

2 A baseline in the interval immediately preceding the go stimulus is problematic if one anticipates in this interval substantial amplitude differences between conditions, such as the switch vs. repeat positivity documented in the task-switching literature (e.g., Elchlepp et al., 2012). Baseline correction is the subtraction of the mean amplitude of the baseline from each time-sample following the baseline. Assuming the difference present during the baseline interval would soon dissipate, this difference would be ‘propagated forward’ later in the go stimulus segment, only with a polarity reversal (a switch-induced positivity during the baseline would result in an apparent, yet entirely artifactual, extended switch-induced negativity later in the ERP segment).

3 To avoid excessive fatigue in our subjects we aimed to keep the duration of the experiment at around 1 h, excluding the EEG preparation procedures. This meant that the trial number in the smallest cell for the ERP analysis (long CSI, no-signal context repeat trials) was 54 at the start. We had previously established that each subjects’ ERP average should contain at least 20 trials. After applying the exclusion criteria ten subjects had less than 20 trials left and were therefore excluded.

4 In this study, it was difficult to correlate the ERP switch-repeat difference with the behavioral switch cost because a smaller behavioral switch-repeat difference could be due to advance preparation on switch trials or increased response slowing (due to response strategy adjustments) on repeat trials.
